# Regulation of ultradian pulsatility and stress responses in the human hypothalamic–pituitary–adrenal axis

**DOI:** 10.1111/jne.70264

**Published:** 2026-09-08

**Authors:** Belinda Lombard, Thomas J. Upton, Ben Gibbison, David Henley, Georgina Russell, John R. Terry, Stafford L. Lightman, Eder Zavala

**Affiliations:** ^1^ School of Mathematics, College of Engineering and Physical Sciences University of Birmingham Birmingham UK; ^2^ Henry Wellcome Laboratories for Integrative Neuroscience and Endocrinology, Translational Health Sciences, Faculty of Health Sciences University of Bristol Bristol UK; ^3^ Bristol Medical School University of Bristol Bristol UK; ^4^ NIHR Bristol Biomedical Research Unit University Hospitals Bristol and Weston NHS Foundation Trust Bristol UK; ^5^ Department of Endocrinology and Diabetes Sir Charles Gairdner Hospital Perth Australia; ^6^ Medical School The University of Western Australia Perth Australia; ^7^ Department of Diabetes and Endocrinology North Bristol NHS Trust Bristol UK; ^8^ Centre for Intellectual Disability Equitable Research (CIDER), Peninsula Medical School University of Plymouth Truro UK; ^9^ Neuronostics Ltd. Bristol UK; ^10^ Centre for Biological Timing, Division of Integrative Physiology, Faculty of Biology, Medicine and Health University of Manchester Manchester UK

**Keywords:** circadian rhythm, ultradian rhythm, stress response pathway, negative feedback, mathematical model

## Abstract

The hypothalamic–pituitary–adrenal (HPA) axis is a vital neuroendocrine system that maintains homeostasis by regulating stress hormone secretion through tightly controlled circadian and ultradian rhythms. Although rodent studies and mathematical models have clarified the mechanisms of ultradian pulsatility, a quantitative, human‐specific description of HPA axis dynamics is lacking. Here, we analyse 24‐h high‐frequency hormonal profiles from a cohort of healthy humans to quantify inter‐individual variability in adrenocorticotropic hormone and cortisol rhythms and to calibrate a foundational mathematical model of the human HPA axis. The model reproduces key human‐specific features, including an asymmetric circadian rhythm peaking at waking and ultradian oscillations with a longer period than those observed in rodents. We show that a simplified representation of the negative feedback between the pituitary and adrenal glands suffices to generate ultradian pulsatility, arising in the model via a Hopf bifurcation. Computational simulations further show how idealised stressors of different intensity, duration and timing perturb HPA axis dynamics, providing a theoretical basis for understanding the heterogeneous stress responses observed in clinical settings. This study provides a quantitative, proof‐of‐concept framework for human HPA axis regulation, offering insights into the potential mechanisms underlying the individual variability and robustness of hormonal rhythms.

## 1 INTRODUCTION

**FIGURE 1:**
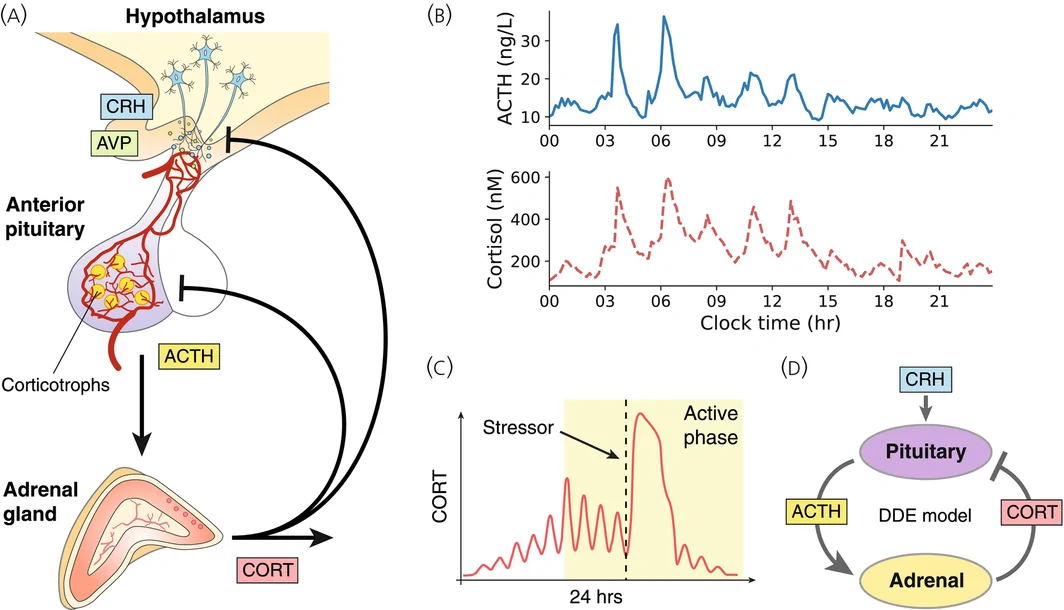
Dynamic regulation of stress hormones. (A) Negative feedback loops within the HPA axis. (B) Ultradian pulsatility of ACTH and CORT with circadian modulated amplitude. (C) HPA hormone rhythms can be temporarily disrupted by stressors. (D) A model of the pituitary–adrenal feedback loop system driven by CRH. Panels (A) to (C) adapted from Galvis et al. and Zavala et al.,^4^, ^5^ under a CC BY 4.0 license.

To maintain good health, our bodies constantly adapt to ever‐changing internal and external conditions through self‐regulating homeostatic processes. One such process is the stress response, a hormonal response to real or perceived stressors that acts to counteract the negative effects of stress. The hypothalamic–pituitary–adrenal (HPA) axis is the neuroendocrine system that regulates the secretion of so called stress hormones such as cortisol (CORT) which reduces inflammation and modulates metabolism, cardiovascular function and cognitive performance.^1^ The HPA axis is dynamically regulated, with circadian and stress inputs processed in the paraventricular nucleus of the hypothalamus, leading to the secretion of corticotrophin‐releasing hormone (CRH) and arginine vasopressin (AVP). CRH stimulates anterior pituitary corticotrophs to release adrenocorticotropic hormone (ACTH). While AVP can also activate ACTH secretion, it does so through a different post receptor signalling mechanism to CRH which results in amplifying the effect of CRH.^2^, ^3^ ACTH then travels through the bloodstream and signals the adrenal glands to release CORT. While CORT can reach all tissues in the body, it can also inhibit ACTH secretion, thus closing a negative feedback loop control mechanism (Figure 1A). In basal, non‐stressed conditions, ACTH and CORT fluctuate throughout the day with an ultradian rhythm (<24 h period) (Figure 1B). The amplitude of these pulses varies circadianly (every 24 h), with a maximum occurring at the time of waking.

The HPA axis can also be thought of as a dynamical system, that is, a system that evolves over time as determined by regulatory processes. As the primary neuroendocrine system responsible for the stress response, the HPA axis is well‐adapted to be highly responsive to a wide variety of stimuli. In non‐stressed conditions, its baseline hormonal rhythms correspond to a state of dynamic equilibrium. Following a stressor, the HPA axis rapidly secretes CORT on top of the endogenous CORT rhythms, with feedback loops helping restore normal rhythmicity once the stressor has ceased (Figure 1C). However, while the body copes well with mild stressors, stronger or longer‐term disruptions may struggle to return to their homeodynamic equilibrium.^6^ This results in misaligned hormonal rhythms, with important implications for clinical care.^4^, ^7^, ^8^ Understanding what makes HPA axis rhythms robust to some perturbations but fragile to others requires a theoretical framework accounting for the origin of ultradian pulsatility of ACTH and CORT, as well as for the disruption of oscillatory behaviour during different types of stress.

Mathematical modelling and experimental physiology have shown that the pituitary–adrenal feedback loop is a key regulatory mechanism behind near‐hourly HPA axis rhythms in rats.^9^, ^10^ In this framework, ultradian oscillations arise from the pituitary–adrenal feedback loop mediated by ACTH and CORT (Figure 1A), with a delay associated with the elapsed time for the information carried by these hormones to flow between the pituitary and adrenal glands. Intuitively, delayed negative feedback generates oscillations because the system responds to deviations from its target state, but only after a time lag. By the time the feedback acts, the system has already overshot its target, so the correction drives it past the target in the opposite direction, with repeated overshooting generating oscillations. This framework contrasts with other neuroendocrine pulse generator mechanisms in that oscillations arise independently of the pulsatility of neuropeptides upstream of the feedback loop.^11^, ^12^, ^13^ Experimental data from multiple species including mice, rats, ewes and monkeys show that CRH is secreted in a pulsatile manner.^14^ While evidence of human CRH pulsatility with a period of approximately 35 min is only available in patients with a disrupted HPA axis,^15^ it is reasonable to assume human CRH is also pulsatile in non‐stressed conditions. However, animal studies also suggest that CRH pulsatility is not the driver of downstream pituitary–adrenal rhythms. Rats with a lesioned suprachiasmatic nucleus or those exposed to constant light for 5 weeks experienced disrupted corticosterone circadian rhythms while their ultradian pulsatile pattern remained unchanged.^16^ In sheep, ultradian rhythms of ACTH and CORT persist after surgical disconnection of the hypothalamus from the pituitary.^17^ Although AVP is involved in both the circadian control of CRH activity and in the activation of corticotrophs, especially during chronic stress,^2^, ^3^, ^18^ CRH is the predominant activator of ACTH secretion, including in response to acute stressors.^19^ In the pituitary–adrenal pulse generator framework, CRH acts as an external input that modulates the amplitude and circadian timing of ultradian ACTH and CORT rhythms, rather than directly generating them. Despite its qualitative nature, the model by Walker et al. was able to predict near hourly pulsatility of corticosterone in rats independently of ultradian oscillations in upstream CRH. Importantly, Walker's model did not attempt to predict dynamic responses elicited by stressors. Despite this progress, a quantitative, human‐specific description of HPA axis dynamics calibrated against high‐frequency sampled data is still lacking.

In this work, we analysed 24 h hormonal time series data collected from high‐frequency blood sampling in healthy humans (*n* = 10).^7^, ^20^, ^21^ We quantified the inter‐individual variability in ACTH and CORT rhythms and used it calibrate a mathematical model of the HPA axis (Figure 1D). The model reproduces dynamic behaviour that is unique to the human HPA axis, namely, an asymmetric circadian rhythm with a maximum at the time of waking and an ultradian rhythm with a period near twice as long than in rodents. Consistent with previous findings,^9^, ^10^ our model predicts that the delayed negative feedback between the pituitary and adrenal glands underpins ultradian pulsatility of ACTH and CORT. In particular, we found that the CRH drive has a modulatory role in ultradian rhythmicity, rather than being part of the core pulse generating mechanism. We discovered that the ultradian rhythm arises through a Hopf bifurcation, that is, a behaviour of certain dynamical systems where changes in parameter values past specific thresholds trigger a switch between oscillatory and non‐oscillatory (steady state) dynamics. Lastly, we performed computer simulations of the model to predict the dynamic responses to stressors of a different intensity, duration and timing. This systematic approach allowed us to make qualitative comparisons with hormonal stress responses observed during and after heart surgery.^4^

## 2 METHODS

### 2.1 Data description and availability

We used published data from dynamic profiles of plasma ACTH and CORT collected from healthy night sleeping individuals. Each participant was sampled over 24 h, with data points spaced every 10 min (*n* = 3)^7^, ^20^ and every 20 min (*n* = 7).^21^ Only data from healthy, unmedicated volunteers were used to calibrate the model. Sex ratio was 9M/1F. Mean age was 37 years, range 24–65 years. Average body mass index was 25.9 ± 2.5 kg/m^2^. Participants maintained conventional work and sleeping patterns and reported no recent (within 10 days) transmeridian travel, weight change less than 2 kg in 6 weeks, shift work, intercurrent psychosocial stress, prescription medication use, substance abuse, neuropsychiatric illness, or acute or chronic systemic disease. A complete medical history, physical examination, and screening tests of haematological, renal, hepatic, metabolic, and endocrine function were normal. No subject had been exposed to glucocorticoids within the preceding 3 months or to estrogen‐containing oral contraceptive medication within the past 6 weeks. Participants were asked to maintain a regular bedtime, abstain from alcohol and all medication, and to avoid any strenuous physical activity for 48 h prior to the sampling session. Ambulation during blood collection was permitted to the lavatory only. Vigorous exercise, daytime sleep, snacks, caffeinated beverages, and cigarette smoking were disallowed. Meals were provided at 08:00, 12:30–13:00, and 17:30–19:00, with a snack available at 22:00. Room lights were turned off between 19:30–24:00 and until 07:00–09:00 depending upon individual sleeping habits. Participants were asked to dim electronic devices and activate “night modes” during these times.

Time series data was standardised by cubic interpolation with uniformly spaced data points (every 1 min for model fitting and every 10 min for wavelet analysis). Metadata on sleep–wake cycles for the participants was not available. As sleep is also regulated by circadian processes and functionally synchronised with hormonal rhythmicity, its absence is noted as a limitation. In the absence of sleep–wake data, the contributory effects of various circadian processes, including sleep‐dependent effects, cannot be separated from the hormonal time series data.

### 2.2 Rhythmometry analysis

We used wavelet analysis to characterise the periodicity of hormonal time series data. This method is based on the Fourier Transform, except it also identifies the position of the frequency in the spatial domain. This allows the mapping of frequency components at specific time points.^22^ Wavelet analysis is particularly useful for analysing noisy oscillatory signals with time‐varying periodicity, which makes it well‐suited to characterise the periodicity of hormonal rhythms. We used pyBOAT, a Python‐based package designed for oscillatory signal analysis, to conduct the wavelet analysis.^23^

Hormonal time series were detrended from the circadian component with a cutoff period of 1440 min (24 h) and normalised. As interpolation does not increase temporal resolution, periodicities below 50 min were excluded from the analysis. The wavelet power of CORT and ACTH profiles of each participant was computed to identify predominant periodicities within a range of 50 to 300 min. We optimised the sliding window width for each participant (mean 765 ± 123 min) by selecting values that provided a stable representation of the dominant ultradian periodicity in the wavelet spectra. We confirmed the robustness of this choice using sensitivity analysis to various window lengths (Figure S9).

To complement the wavelet analysis, we performed cosinor‐based fitting and periodogram analysis on the circadian‐detrended CORT profiles.^24^, ^25^ Models allowing for secondary oscillatory modes were fitted across a range of plausible ultradian periods (50–250 min). The best‐fitting period for each model was identified by adjusted *R* ^2^, and the simpler model was retained unless an *F*‐test justified the additional modes.

### 2.3 Mathematical model

We simplified the model by Malek et al.^26^ to account for a delayed pituitary–adrenal feedback loop underpinning the dynamics of ACTH and CORT. Our model does not consider the hypothalamic feedback loop because the anterior pituitary is known to be a major site for glucocorticoid feedback^27^ and because of the slow CRH response following glucocorticoid stimulation.^28^ The model is written in terms of delay differential equations (DDEs) and is given by:

$$\frac{\mathrm{dA}}{\mathrm{dt}}=-\gamma_{a}A+\frac{K_{c}^{m_{a}}\Phi \left(t\right)\;}{K_{c}^{m_{a}}+C{\left(t-\tau \right)}^{m_{a}}}$$

$$\frac{\mathrm{dC}}{\mathrm{dt}}=-\gamma_{c}C+\alpha \frac{A^{m_{c}}\;}{K_{a}^{m_{c}}+A^{m_{c}}}$$

where A and C accounts for ACTH and CORT, respectively. The delay τ represents the time that it takes for CORT to inhibit the release of ACTH since it is been secreted. This delay therefore captures the cumulative elapsed time of multiple physiological processes such as hormone synthesis and secretion, transport via the circulation, receptor binding, and intracellular glucocorticoid feedback. The Hill‐type functions represent nonlinear interactions, capturing saturation and sensitivity in hormone receptor‐mediated signalling. The Hill coefficients describe the steepness of these responses, not specific molecular mechanisms. The model development is detailed in Data S1, with the remaining model parameters described in Table S1. We adopt the pituitary–adrenal pulse generator framework as a modelling hypothesis for humans, with CRH acting as an amplitude modulator of ACTH and CORT pulsatility.^9^, ^10^ Therefore, we represent CRH as an external drive function $\Phi \left(t\right)$ which could be either constant or have a circadian profile given by:

$$\Phi \left(t\right)=\lambda_{a}e^{\lambda_{s}\cos\left(2\pi \;\frac{t-t_{s}}{T_{c}}+\sigma \cos\left(2\pi \;\frac{t-t_{s}}{T_{c}}\right)\right)}$$

where the circadian period $T_{c}$ is fixed at 1440 min. We introduce the parameter σ to account for the observed asymmetry in the circadian profile of ACTH and CORT in humans,^19^ while $\lambda_{s}$ and $\lambda_{a}$ account for the variability in the amplitude of the circadian rhythm. Similarly, we introduce parameter $t_{s}$ to account for the heterogeneity in the circadian phase of individuals within the cohort. Our model assumes that CRH primarily modulates system gain without requiring autonomous pulsatile activity. As an external drive, CRH therefore acts as a hypothalamic modulatory input rather than being part of the oscillatory core.

### 2.4 Parameter estimation

We estimated parameter values that best fit ACTH and CORT time series data from healthy participants using the open‐source Python library Probabilistic Inference for Noisy Time Series (PINTS) with the Covariance Matrix Adaptation Evolution Strategy fitting procedure.^29^ The DDE model was solved using the Python ddeint solver, with the cost function defined as the sum of least squares between normalised ACTH and CORT signals. Parameters were fitted to each participant's data and constrained within physiologically plausible ranges (Table S1), yielding personalised sets that best reproduced specific dynamic behaviours within each participant: ultradian period, phase lag between ACTH and CORT, circadian dynamic range, circadian asymmetry, and time of the circadian peak. During model calibration, only solutions having between 5 and 14 pulses per 24 h were accepted, corresponding to ultradian periods between 100 and 288 min, as identified by the wavelet analysis. Parameter distributions were calculated to determine confidence intervals for model robustness and to derive cohort‐level parameter estimates.

Given the number of model parameters relative to the cohort size, we acknowledge the potential for challenges in parameter identifiability (i.e., different parameter combinations may lead to similar model outputs). The reported parameter distributions (Figure 3B) represent the set of values that provided the best fit under the constraints of our model structure and should be interpreted as effective parameters capturing the observed dynamics for this cohort, rather than uniquely identifiable physiological quantities. To evaluate model generalisability, we used leave‐one‐out cross‐validation (LOO‐CV). For each participant, we used the mean model parameters from the remaining nine individuals and simulated their hormone time series after adjusting $t_{s}$. We calculated prediction error as the normalised sum of squared errors and summarised generalisation performance as the ratio of LOO‐CV error to participant fit error.

### 2.5 Bifurcation analysis

We used DDE‐BIFTOOL,^30^ a MATLAB package for bifurcation analysis of DDEs, to perform numerical continuations of our model and sketch two‐parameter bifurcation diagrams that defined regions where self‐sustained oscillatory solutions exist. We chose a constant CRH drive Φ and the pituitary–adrenal delay τ as bifurcation parameters. Continuations were performed with a step size of 0.01, with maximum parameter steps of 0.5 for the delay τ and of 10 for the CRH input Φ. Each branch was continued for up to 1400 steps, with a Newton tolerance of $10^{-6}$.

### 2.6 Computer simulations of stressors

We simulated an acute stressor as a transient pulse function $S\left(t\right)$ added to the CRH drive function $\Phi \left(t\right)$ upstream the pituitary–adrenal feedback loop. In the absence of CRH data, $S\left(t\right)$ is a qualitative representation of a perturbation of the hypothalamic drive intended to explore possible stress‐induced dynamics. The stressor was represented by the function:

$$S\left(t\right)=\left\{\begin{array}{l} \;0,\;t<t_{s}\;\text{or}\;t\geq t_{s}+t_{r}+t_{d}\\ \;M\frac{t-t_{s}}{t_{r}},\;t_{s}\leq t<t_{s}+t_{r}\\ Me^{-k\frac{\left[t-\left(t_{s}+t_{r}\right)\right]}{t_{d}}},\;t_{s}+t_{r}\leq t<t_{s}+t_{r}+t_{d} \end{array}\right.$$

which describes of a transient ($t_{r}=10$ min) rise in CRH at time $t_{s}$, followed by an exponential decay once the stressor has ceased. This paradigm is typical of sudden, short‐lived stress, as opposed to slow‐building and chronic stress. Four parameters govern the stressor dynamics: magnitude M (size of the CRH excursion), duration $t_{d}$ (elapsed time to decay back to baseline), time $t_{s}$ (when the stressor occurs), and k as a decay rate constant. These parameters allowed us to systematically adjust the shape of the stressor function and predict its dynamic effects on ACTH and CORT.

The stressor function was modified to account for the longer, sustained stress elicited by heart surgery.^4^ This included a rising phase that saturates at peak magnitude M, followed by a plateau of varying length, and an exponential decay. This is given by:

$$S_{\alpha ,\beta }\left(t\right)=\left\{\begin{array}{l} \;0,\;t<t_{s}\\ \;M\frac{1-e^{-\rho \frac{t-t_{s}}{t_{r}}}}{1-e^{-\rho }},\;t_{s}\leq t<t_{s}+t_{r}\\ \;M,\;t_{s}+t_{r}\leq t<t_{s}+t_{r}+t_{p}\\ \;M\;e^{-\kappa {\left[t-\left(t_{s}+t_{r}+t_{p}\right)\right]}^{\beta }},\;t\geq t_{s}+t_{r}+t_{p} \end{array}\right.$$

where $t_{s}$ is the onset, $t_{r}$ the rise duration, $t_{p}$ is the duration of a plateau following the rise and M the stressor magnitude. The exponential terms are ρ>0 which determines the speed of the initial increase, κ>0 which sets the overall decay rate and β>0 which controls the decay rate.

By varying these parameters, we generated stressors with (i) steep rises and fast decays (ii) long increases with slow returns to baseline, and (iii) sustained plateaus to represent extended surgical stress (see Data S1).

## 3 RESULTS

### 3.1 Variability of ultradian rhythms in human HPA axis hormones

**FIGURE 2:**
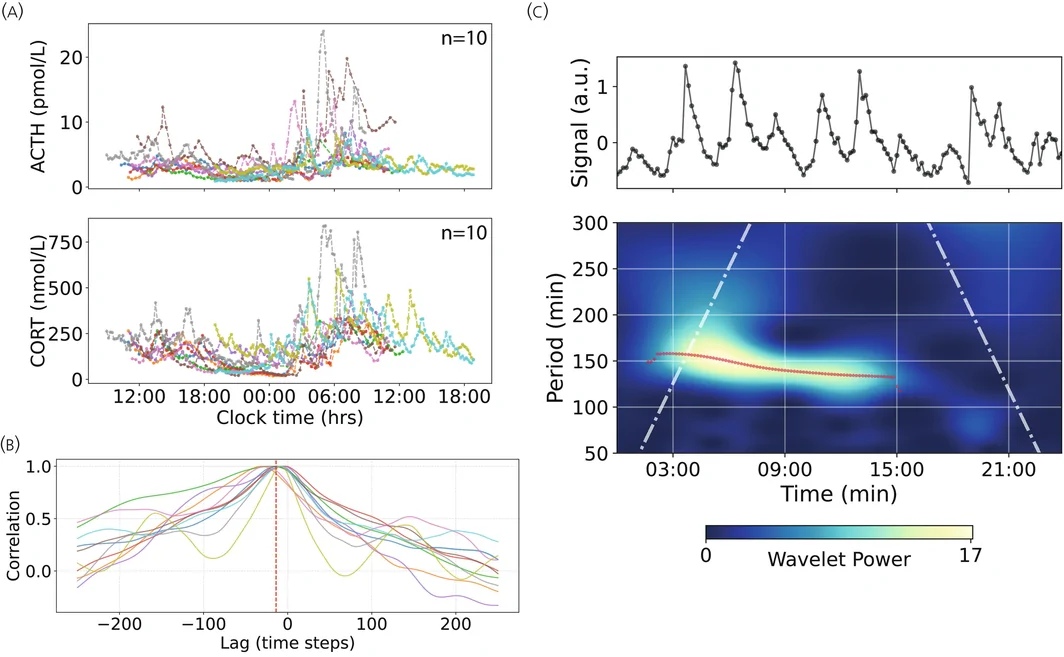
Rhythmic secretion of (A) ACTH and CORT in healthy humans (*n* = 10) over 24 h, measured every 10 min (*n* = 3) and every 20 min (*n* = 7). (B) Time‐lagged cross‐correlations between ACTH and CORT showed negative lags for all participants, indicating ACTH precedes CORT by 13.8 ± 8.6 min (red dashed line). (C) Wavelet analysis of CORT rhythms for a single participant. Top: circadian‐detrended time series. Bottom: periodogram quantifying ultradian periodicity over time. White dotted‐dash line: cone of influence of boundary effects. Red dotted line: peaks of dominant periodicity. The variability in the ultradian band over time may reflect physiological factors and methodological limitations due to the relatively short time series with respect to the ultradian period and lower signal to noise ratio in the evening. See Figure S2 for the other participants.

We examined 24‐h profiles of plasma ACTH and CORT in healthy participants (*n* = 10) and found regular pulsatile secretion indicative of ultradian rhythmicity (Figure 2A). The amplitude of these pulses showed asymmetric circadian modulation, rising overnight for ~8 h with a morning peak and falling throughout the day for ~16 h with a nadir in the evening.

To quantify the synchronicity between ACTH and CORT, we computed the time‐lagged cross‐correlation between hormone pairs for each participant (Figures 2B and S1). Consistent with previous findings,^21^ we estimated that on average CORT lags behind ACTH by 13.8 ± 8.6 min. To characterise the ultradian periodicity, we applied wavelet analysis on circadian‐detrended hormonal signals (Figure 2C). Periods with the highest wavelet power ranged between 100 and 250 min across all participants, confirming the presence of ultradian rhythmicity. This period range was robust to variation in the wavelet sliding window length, including when no signal normalisation was applied (Figure S9). We found that most participants (*n* = 6) had a single dominant ultradian period, whereas the remaining participants displayed smaller secondary peaks in the wavelet power spectrum (Figures S2, S3), suggesting the possibility of additional oscillatory modes. Cosinor analysis further showed that secondary periodicities could achieve comparable cosinor model fits in some participants, making it difficult to identify a single dominant period (Figures S10, S11).

### 3.2 A pituitary–adrenal feedback loop reproduces the heterogeneous rhythmic behaviour of the human HPA axis

**FIGURE 3:**
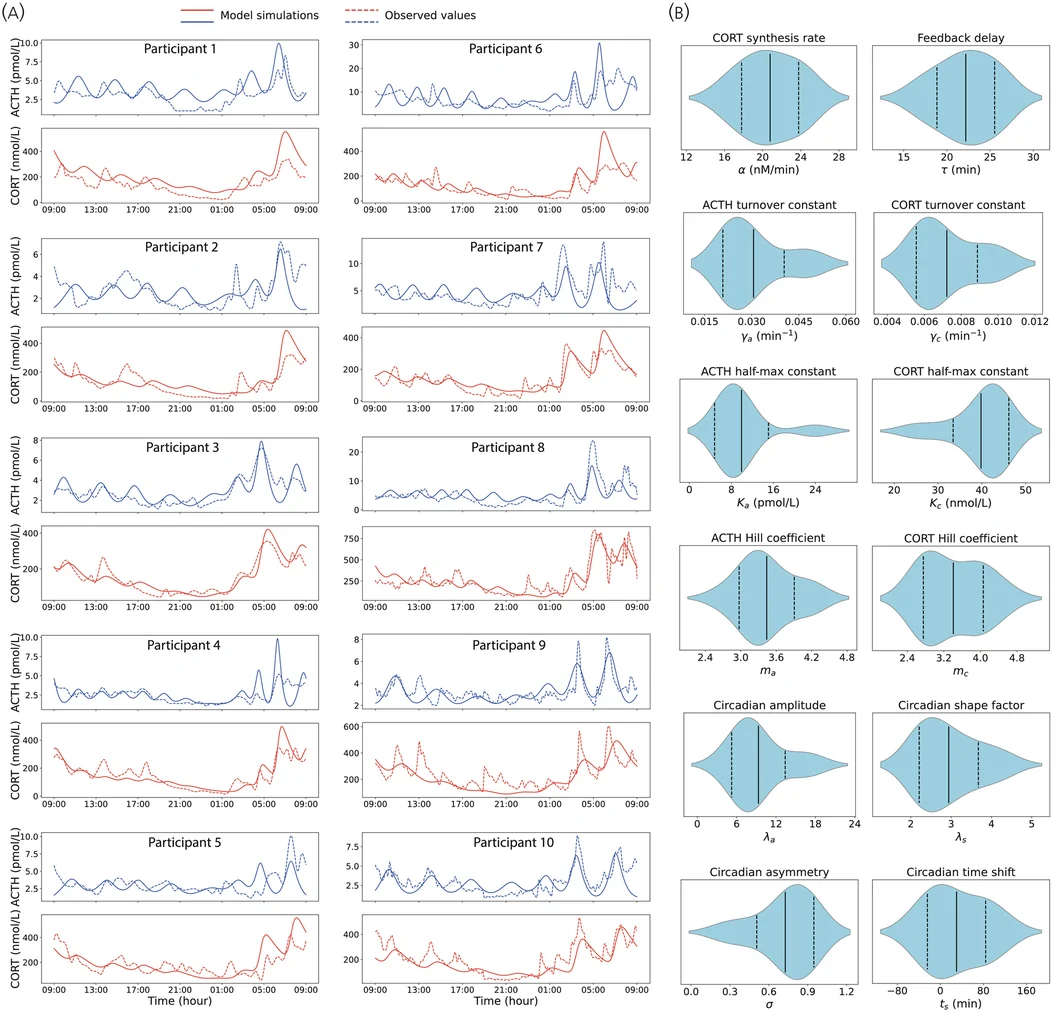
Model calibration and parameter estimation. (A) Best model predictions of ACTH and CORT rhythms in healthy humans (*n* = 10). Simulations were obtained by fitting model parameters individually to each participant's data. (B) Distributions of parameter values that best fit the cohort data, including those that shape the circadian drive ($\lambda_{a}$, $\lambda_{s}$, σ, $t_{s}$), Hill coefficients (*m*_*a*_, *m*_*c*_), feedback sensitivities ($K_{a}$, $K_{c}$), synthesis and turnover rates (*α*, *γ*_*a*_, *γ*_*c*_) and the feedback delay (τ). Table S1 lists median values and variation for each parameter, along with their physiological interpretation.

To investigate whether the variability of hormonal rhythms across all participants could be explained by the same regulatory architecture within the HPA axis, we developed a mathematical model of the pituitary–adrenal feedback loop. The model reproduced the dynamics of ACTH and CORT, including modulation from a hypothalamic CRH drive (Figure 1D), and was individually fitted to each participant in the cohort. The model reproduced the ACTH and CORT rhythms observed in healthy participants under non‐stressed conditions, including ultradian periodicity and asymmetric circadian modulation (Figure 3A). Computer simulations reproduced individual differences in the amplitude, frequency and phase of ultradian pulsatility, as well as their asymmetric circadian modulation. We estimated the distributions of fitted parameter values, which quantified the variability of the cohort within physiologically plausible values (Figure 3B). Both parameters governing the feedback loop (Hill coefficients $m_{a}$, $m_{c}$ and feedback sensitivities $k_{a}$, $k_{c}$) as well as the circadian drive (*λ*_*a*_, *λ*_*s*_, *σ*, and *t*_*s*_) exhibited inter‐individual differences, reflecting physiological differences in the feedback strength, adrenal and pituitary sensitivity, and circadian modulation of the HPA axis.

To assess model generalisability, we used LOO‐CV, where estimated parameters from a cohort of nine participants predicted the left‐out individual. LOO‐CV prediction errors were moderately higher than individual fits (mean ratio ~1.5). The model accurately reproduced dynamic behaviour across individuals despite variability, with the cohort model even outperforming the individual fit in 2 out of 10 cases (Figures S12, S13 and Table S2). This suggests that our model captures a shared dynamical structure, rather than relying on highly specific parameter combinations for each individual.

### 3.3 A pituitary–adrenal feedback loop underpins self‐sustained ultradian rhythms of ACTH and CORT

**FIGURE 4:**
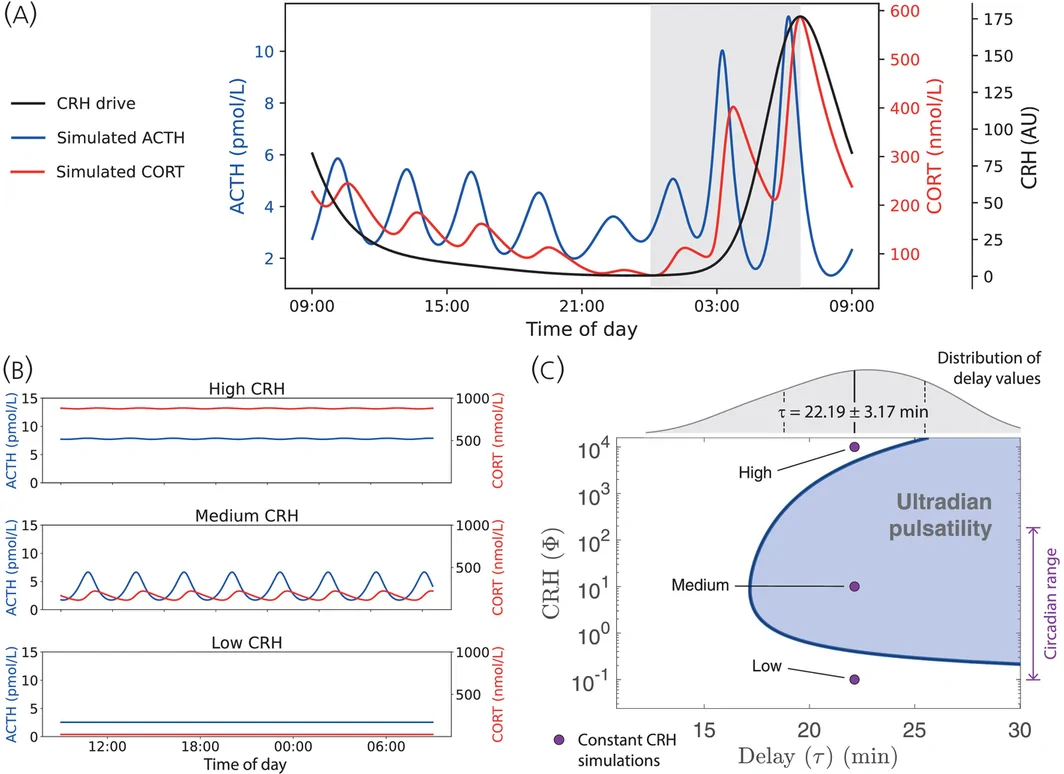
Mathematical model predicts ultradian pulsatility. (A) ACTH and CORT dynamics predicted by the model using best fit mean parameter values for the cohort (*n* = 10) and an asymmetric circadian CRH drive. (B) Predicted ACTH and CORT dynamics under low, medium and high constant CRH (0.1, 10 and 10,000 AU, respectively) with fixed delay at τ = 22.19 min. (C) Two‐parameter bifurcation diagram predicting ultradian pulsatility (blue‐shaded region) occurs for specific combinations of CRH values and feedback delay τ.

Next, we investigated the conditions under which the pituitary–adrenal feedback loop could sustain ultradian rhythms of ACTH and CORT, and the ability of the model to account for inter‐individual variability. To do this, we performed computer simulations using mean parameter values from their estimated distributions (Figure 3B), therefore constituting a model representative of the cohort. This population model accurately reproduced key features of the HPA axis dynamics, including ultradian rhythms of ACTH and CORT with a period of approximately 2.5 h, with ACTH phase advanced relative to CORT (Figure 4A). Additionally, the amplitude of ultradian pulses exhibited an asymmetric circadian profile, with an evening nadir around midnight and a morning peak just after 07:00 h. The model also predicted hormonal concentrations within their physiological dynamic range in blood in non‐stressed conditions.^20^, ^21^

Following previous work,^10^ we removed the time‐dependent circadian drive and simulated the population model under constant CRH at low, intermediate and high input levels (0.1, 10 and 10,000 AU, respectively). We found that constant low and high CRH levels led to, respectively, constant low and high ACTH and CORT concentrations. In contrast, intermediate, constant CRH stimulation led to self‐sustained ultradian oscillations of ACTH and CORT (Figure 4B). These results suggest that ultradian rhythms of ACTH and CORT in humans can emerge in the absence of circadian cues, consistent with previous findings from mathematical and rat models.^9^, ^10^, ^16^ To disentangle the origin of ultradian rhythms in ACTH and CORT, we simulated the effect of pulsatile CRH on the system dynamics at different periods (35, 60 and 90 min) and baseline CRH levels (Figure S8). A pulsatile CRH drive at low and high baselines forced ACTH and CORT to follow the imposed CRH oscillatory period. At medium CRH levels, however, the model predicts that ACTH and CORT preserve the intrinsic ultradian periodicity reported in humans despite oscillatory CRH forcing at different frequencies. Interestingly, simulations of a pulsatile CRH drive with 90 min periodicity (near the lower bound of the ultradian period range observed in humans) indicate stronger modulation in the timing and amplitude of ACTH and CORT oscillations, suggesting that CRH pulsatility could still act as a modulator, but not the main driver of ultradian ACTH and CORT rhythms.

We performed a two‐parameter bifurcation analysis to determine the combination of CRH inputs Φ and feedback delay τ for which the model predicts rhythmic solutions. We found ultradian oscillations exist only within a bounded parametric region with high enough feedback delay values and intermediate CRH inputs as determined by the bifurcation diagram in Figure 4C. All other parameters remained fixed at their mean values. Consistent with previous findings,^10^, ^13^ our analyses show that ultradian oscillations emerge via a Hopf bifurcation (Figure S4), with the corresponding limit cycle solution associated to a dynamic equilibrium.

We also examined how inter‐individual differences in parameter estimates mapped onto their individual bifurcation diagrams (Figure S5). Most individuals (*n* = 7) operated completely within the oscillatory region, while the rest (*n* = 3) had parameter estimates where they only marginally left and re‐entered the bifurcation region.

### 3.4 The model reproduces hormonal dynamic responses to heart surgery

**FIGURE 5:**
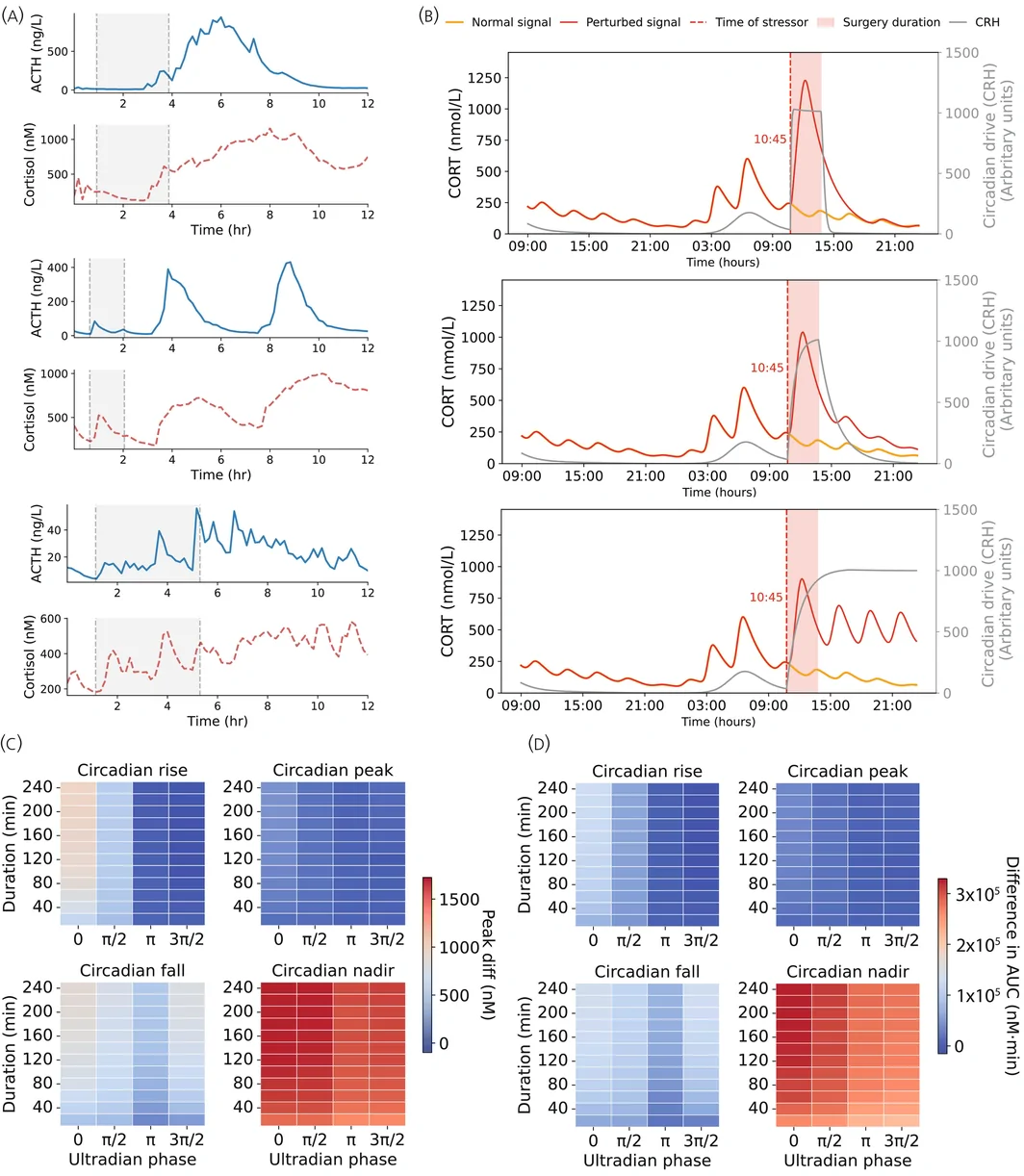
Heterogeneity of dynamic responses to surgery stress. (A) Examples of dynamic responses of the HPA axis to heart surgery, showing distinct dynamic phenotypes including a single pulse (top), two pulses (centre), and sustained pulsatility (bottom) of ACTH and CORT hormones. Reproduced from^4^ under a CC BY 4.0 license. (B) Computer simulations of dynamic responses to heart surgery. The stressor is modelled as a CRH impulse disrupting the circadian drive. Predicted CORT profiles after the stressor (red) are compared versus the non‐stressed dynamics (blue). Each panel shows the dynamic effect of varying the shape of the simulated stressor (grey). From top to bottom: a plateau stressor with rapid onset and decline results in a transient CORT surge followed by a slow rhythm recovery; a stressor with a gradual rise and decline leads to a quicker recovery of ultradian rhythmicity; a prolonged stressor leads to CORT pulsatility at a high baseline. (C) Phase‐response heatmaps showing CORT peak surges and (D) AUC changes as a function of the timing and duration of the stressor while keeping its amplitude fixed (150 AU). The ultradian phase is described in radians as 0 (nadir), *π*/2 (rising), π (peak), and 3π/2 (falling).

To test whether the model could reproduce dynamic responses to stressors in a qualitative yet physiologically meaningful way, we simulated the stress response to heart surgery. As a case study, we used our previous findings where CORT responses to coronary artery bypass grafting (CABG) were reported at high time resolution.^4^ Despite their heterogeneity, these responses can be grouped into three distinct dynamic phenotypes: those showing a single, large hormonal excursion; those showing ultradian pulses with an increased period and amplitude; and those that preserved near normal ultradian periodicity at an elevated baseline (Figure 5A). We hypothesised that these dynamic phenotypes may be explained by the specific way the stressor perturbs the system away from its dynamic equilibrium (i.e., the limit cycle solution). To test this, we simulated the system under various CRH stimuli that differed only in the shape of their associated stressor function, while keeping invariant both the amplitude and timing of the stressor (see Data S1). We found that a sustained CRH stimulus with a plateau stage lasting for the duration of surgery elicits a large CORT surge that smoothly returns to normal rhythmicity (Figure 5B, top). In contrast, a CRH stimulus that gradually builds up during the surgery before exponentially decaying elicits a smaller CORT increase followed by ultradian pulses during the falling phase (Figure 5B, centre). Lastly, a CRH stimulus that gradually builds up during surgery and remains elevated at a constant plateau beyond the end of the procedure results in a CORT increase followed by self‐sustained ultradian oscillations at a baseline determined by the CRH value at the plateau (Figure 5B, bottom). Altogether, these results suggest that the dynamics of the disruption on the hypothalamic drive elicited by a stressor will impact the dynamic behaviour of CORT during the stress response.

### 3.5 The dynamic stress response is sensitive to the timing of the stressor

Lastly, we used the model to interrogate HPA axis responses to acute stressors of varying duration and timing (i.e., phase or time at which they arrive) while keeping their magnitude invariant (CRH drive fixed at 150 AU). We investigated phase effects by simulating four different time points at which the stressor occurred, corresponding to distinct phases of the circadian cycle: peak (~07:30 h), decline phase (~13:00 h), nadir (~00:00 h), and rising phase (~03:00 h). Within each circadian phase, stressors were simulated across four ultradian phases (peak, decline, nadir, and rise) (Figure S6). In all cases, the resulting stress response was quantified via phase response heatmaps describing (i) the difference in CORT concentration between the perturbed and unperturbed signal at the first peak following the stressor (Figure 5C), and (ii) the difference in the area under the curve (AUC) between the perturbed and unperturbed CORT signals over 24 h after the stressor was introduced (Figure 5D).

When the stressor magnitude was kept constant, we found that the system is the most sensitive to perturbations (i.e., have larger deviations from the dynamic equilibrium) during the nadir of the circadian phase (Figure 5C,D). In contrast, the system is least sensitive when the stressor occurs at the peak of the circadian phase. The ultradian phase had a minimal impact on these sensitivities, with only a slightly higher response to the stressor around the ultradian nadir and rising phases. However, stressors occurring during the circadian rising phase resulted in a higher peak and AUC difference in CORT during the ultradian nadir and rising phases than during the peak and falling phases. Lastly, during the circadian falling phase, stressors arriving during the ultradian peak resulted in a lower peak and AUC difference in CORT with respect to any other ultradian phases. In all cases, increasing the duration of the stressor amplified the differences in peak and AUC CORT, but this effect vanished for stressors longer than 1 h. We also examined the case where stressor duration was fixed at 100 min, but varied in magnitude and timing. The corresponding phase response heatmaps (Figure S7) were similar to the case where the stressor magnitude was fixed (Figure 5C,D). Altogether, this suggests that the time of day at which a stressor arrives is as important as is its duration and magnitude.

## 4 DISCUSSION

In this study, we developed and analysed a mathematical model of the HPA axis calibrated to human data. By using high‐frequency hormone measurements from blood plasma in healthy participants^21^ we have shown that the model can reproduce key features of circadian and ultradian rhythms of ACTH and CORT. The model considers individual variability in the properties of HPA axis rhythms, as quantified by the estimated unimodal distributions of parameter values. We used the model to investigate how dynamic stress responses depend on the timing, duration and intensity of stressors, including hormonal responses reported in heart surgery patients.^4^ It is worth noting that differences in parameter values represent aggregated physiological processes (e.g., feedback strength, adrenal sensitivity, circadian modulation) rather than uniquely identifiable biological quantities. Furthermore, the ability of the model to reproduce a range of dynamic behaviours within the cohort doesn't imply the pituitary–adrenal pulse generator framework is the sole source of variability. Instead, our findings constitute a plausible mechanistic explanation within the modelling framework and should be viewed as a hypothesis‐generating tool.

Our results are consistent with previous findings from mathematical modelling and experimental physiology in rats that a delayed negative feedback between the pituitary and adrenal glands is sufficient to generate ultradian pulsatility in ACTH and CORT.^9^, ^10^ The period of these ultradian rhythms in humans is between 2 and 3 times longer than in rats, and their amplitude varies circadianly with an asymmetric profile that increases overnight, peaks at the time of waking, and decreases progressively throughout the day. These features were accurately captured by our model, with parameter estimations revealing how the dynamic behaviour of the HPA axis varies across individuals. Additional simulations with pulsatile CRH inputs show that, while ACTH and CORT can track the imposed CRH pulsatility, the intrinsic ultradian rhythm arising from the pituitary–adrenal pulse generator supersedes these imposed dynamics. This suggests that pulsatile CRH may modulate, but is not the main determinant, of the observed ultradian dynamics in ACTH and CORT.

The presence of weakly powered secondary peaks in some wavelet spectra reflects the challenges of analysing short (24 h), discretely sampled (10–20 min), noisy time series data. While choices during interpolation and analysis settings can influence spectrograms, our sensitivity analyses show that the identified ultradian period range is robust to wavelet window length and normalisation. We interpret the additional peaks observed in the wavelet spectra as likely arising from individual variability in pulse timing and amplitude, rather than reflecting the presence of multiple underlying oscillators. By fitting the model to hormonal data, we interrogate what parameter values would reproduce rhythmic behaviour (if at all) within the observed amplitude, phase and period properties. For the latter, we only asked what parameter value estimates would match ultradian periodicity within the physiologically plausible range identified by the wavelet analysis, rather than enforce agreement. The structural simplicity of our mathematical model gives origin to a single‐period self‐sustained ultradian rhythm via delayed negative feedback, and it is not intended to account for multiple oscillatory modes. In short, the model should be interpreted as a minimal, hypothesis‐generating framework that captures observed dynamics, rather than a final representation of the full physiological system.

**FIGURE 6:**
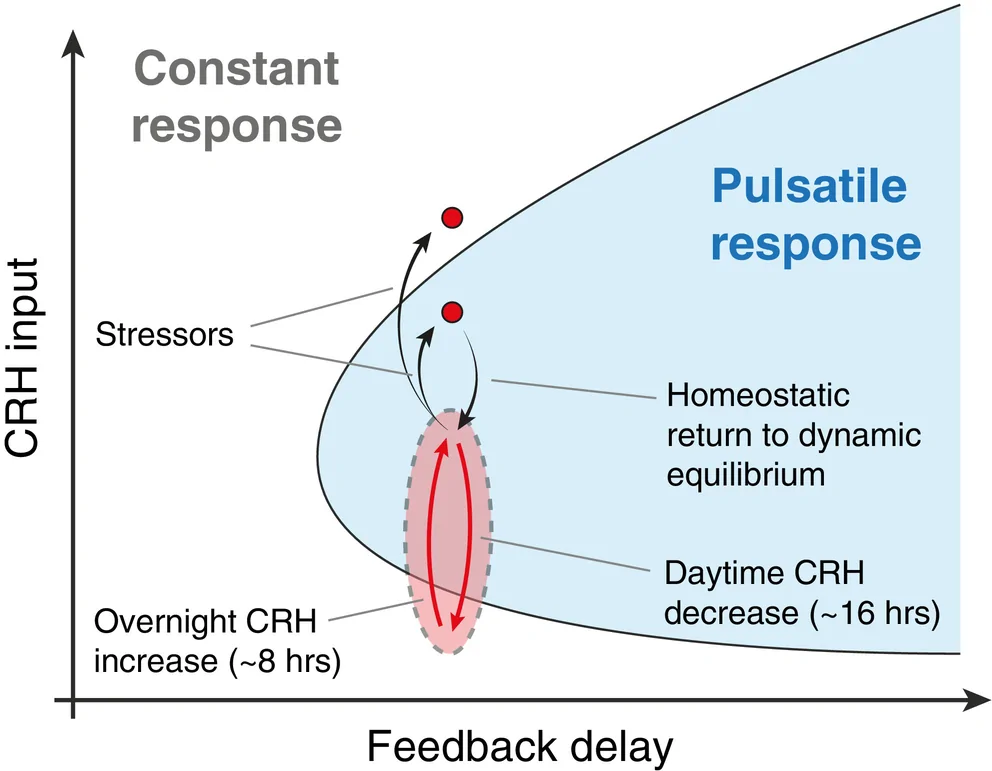
Schematic of how the pituitary–adrenal feedback loop helps maintain a homeostatic dynamic equilibrium of ultradian pulsatility, which can be temporarily disrupted by stressors.

Bifurcation analyses revealed a boundary between oscillatory and non‐oscillatory behaviour determined by the strength of the hypothalamic CRH input and the pituitary–adrenal delay. Since CRH has a circadian rhythm, the model implies the system's operational point moves inwards and outwards of the oscillatory region with circadian periodicity, with an approximately 8 h long overnight increase and a 16 h long daytime decrease (Figure 6). Our parameter estimations suggest the CRH dynamic range and feedback delay at which the circadian drive operates are individual specific, with most individuals' operational point moving well inside the oscillatory region. While the dynamics of some individuals takes place near the bifurcation boundary and, in some cases briefly crossing it, they still mostly operate within the self‐sustained oscillatory region. These findings suggest that inter‐individual variability may be associated to the proximity to the oscillatory boundary, potentially affecting the robustness of ultradian rhythmicity in response to perturbations. Taken together, our findings highlight the potential role of the circadian drive and feedback delay in influencing individual variability. We suggest our model could be used to disentangle the effect of circadian rhythm modulators (e.g., sleep) on HPA axis dynamics across subpopulations.

Our modelling framework can also assess variability to stress responses. Specifically, we interrogated to what degree the heterogeneity in the stress responses to heart surgery could be attributed to the dynamic characteristics of the stressor. The fact that the same model structure is capable of qualitatively reproducing the range of observed dynamic responses to heart surgery, without the need to modify the model assumptions, is testimony of its predictive power. To systematically characterise the dynamic behaviour of CORT during the stress response, we simulated stress perturbations of different magnitude, duration and occurring at different times of the day. This allowed us to explore the robustness of the pituitary–adrenal system as a regulatory system for stress homeostasis. Systematic simulations of stressors showed that both circadian and ultradian phase strongly influence the size and shape of the hormonal stress response. Perturbations applied at the circadian nadir or ultradian troughs resulted in much larger responses than those applied during peak phases. These results suggest that, within our modelling framework, significantly affect hormonal rhythms,^8^ potentially explaining the various dynamic phenotypes observed in patients undergoing heart surgery.^4^, ^31^ Although phase‐dependent stress responses have been reported in animal studies,^32^, ^33^, ^34^, ^35^ and translational studies in humans have shown similar results,^36^ our in silico predictions for humans should be interpreted as qualitative insights rather than direct physiological effects.

To our knowledge, this work is the simplest theoretical framework of the human HPA axis that can simultaneously reproduce ultradian rhythms, circadian asymmetry, individual variability and dynamic responses to stressors. Our estimated parameter value distributions suggest we can quantify individual variability in the system dynamics, including due to factors exogenous to the pituitary–adrenal feedback architecture, such as the circadian drive. The strong phase dependency of the stress responses highlights the importance of considering internal timing with respect to ultradian and circadian rhythms when interpreting hormonal time series data or designing clinical interventions. For instance, the model suggests that the same stressor may elicit different responses depending on whether it occurs at a hormonal peak or trough. Although our study provides a foundational, human‐specific model of the HPA axis, its limitations must be carefully considered when interpreting the results. These limitations, outlined below, also highlight important avenues for future research.

### 4.1 Model reaches, limitations and future directions

#### 4.1.1 Model scope and structure

The model architecture is simple by design, focusing on the minimal components required to generate ultradian pulsatility. Consequently, it ignores several known regulatory pathways. First, the model excludes direct hypothalamic feedback and the modulatory effect of AVP on CRH signalling. In many chronic clinical states, such as depression or chronic stress, evidence suggests that alterations in hypothalamic function and AVP secretion are critical,^37^, ^38^ and our model in its current form cannot capture these dynamics. The lack of sleep–wake cycle data prevents us from disentangling sleep‐dependent effects from hormonal time series data. Accordingly, the circadian drive in our model is solely a representation of the 24‐h CRH modulation of HPA axis activity, rather than a direct measure of intrinsic circadian regulation. The asymmetry in our circadian drive function is therefore a phenomenological fit to the data, and its precise biological basis remains to be determined. Second, our stressor simulations assume that perturbations act solely via a CRH impulse. This is an idealisation, as physiological stressors like surgery involve complex inflammatory and sympathetic responses that can modulate adrenal sensitivity and other aspects of the axis directly.^39^ Third, we do not consider intra‐adrenal regulation, which can also shape the cortisol secretory profile.^40^

#### 4.1.2 Data and parameterisation

A primary limitation is the small cohort size (*n* = 10), which restricts the generalisability of our findings. Because the model was calibrated to healthy participants under controlled conditions, its predictions may not reflect the variability seen in broader populations or in the more complex context of day‐to‐day living. The estimated parameter distributions therefore should be seen as characteristic of this specific cohort of healthy individuals, not as a definitive representation of the broader human population. While the cohort size is small, LOO‐CV indicates that the model generalises well across individuals, with moderate increases in prediction error reflecting inter‐individual variability rather than overfitting. LOO‐CV suggests that the model captures a shared structure across individuals, but this does not guarantee that individual parameters are uniquely identifiable. Different parameter combinations can lead to similar hormonal dynamics, so the inferred parameters should be considered effective values rather than direct physiological quantities. While LOO‐CV alone cannot resolve the issue of parameter identifiability, a comprehensive structural and practical identifiability analysis was beyond the scope of this initial study but will be essential in future work.

#### 4.1.3 Clinical translation

While we make qualitative comparisons to surgical stress responses, we have not validated the model against data from patients with a dysregulated HPA axis (e.g., Cushing's syndrome, adrenal insufficiency) or linked the predicted dynamic phenotypes to clinical outcomes. Therefore, the model should be viewed as a hypothesis‐generating tool. The present theoretical framework could be used to test scenarios of acute, repetitive or chronic stress, particularly clinically relevant perturbations originated from sleep disruptions, surgery (with particular attention to time of day), physical trauma,^41^ and diagnostic tests (e.g., Synacthen and dexamethasone suppression tests). A key modelling challenge will be to consider the role of hypothalamic feedback in the homeostatic return to the dynamic equilibrium following a stressor. Generalisation of the model at longer time scales could account for gland dysfunction in the progression of endocrine disease, leading to dynamic compensation phenomena^42^ and disrupted hormonal rhythms such as those observed in Cushing's^43^, ^44^ and adrenal insufficiency.^45^ Accounting for inter‐ and intra‐individual variability would require calibrating the model to data from larger and more diverse cohorts, while also considering the cross‐talk interactions with other endocrine systems that may impact the HPA axis dynamics such as the metabolic^46^ and reproductive axes.^47^ Integration of novel multimodal data from wearables with mechanistic models could accelerate the development of digital endocrinology applications in precision medicine.^21^, ^48^

#### 4.1.4 Future directions

Our human‐specific model of the HPA axis provides a theoretical framework for understanding hormonal variability at the dynamic equilibrium and homeostatic regulation of the stress response. It also lays the groundwork for several important extensions. The immediate next steps should involve validating and refining the model with data from larger, more diverse cohorts, including participants with known HPA axis pathologies. Incorporating sleep data is a high priority to create a more mechanistically complete model of circadian regulation. Future iterations of the model could also explicitly include the effects of AVP, hypothalamic feedback loops, and direct adrenal modulation to capture a wider range of physiological and pathophysiological states. Ultimately, connecting the model's dynamic predictions to clinical outcomes will be the key to unlocking its potential for precision medicine.

## AUTHOR CONTRIBUTIONS

**John R. Terry:** Writing – review and editing. **Thomas J. Upton:** Writing – review and editing; resources. **David Henley:** Resources. **Ben Gibbison:** Resources. **Belinda Lombard:** Conceptualization; investigation; writing – original draft; methodology; visualization; writing – review and editing; formal analysis; data curation; software. **Eder Zavala:** Conceptualization; investigation; funding acquisition; writing – original draft; project administration; supervision; writing – review and editing; visualization; methodology. **Stafford L. Lightman:** Writing – review and editing; resources. **Georgina Russell:** Resources.

## CONFLICT OF INTEREST STATEMENT

Stafford L. Lightman is co‐founder of Dynamic Therapeutics Ltd. John R. Terry is co‐founder and managing director, and holds equity in Neuronostics Ltd. The remaining authors declared that this work was conducted in the absence of any commercial or financial relationships that could be construed as a potential conflict of interest.

## Supporting information


**Table S1.** Model parameters.
**Table S2**. Leave‐one‐out cross‐validation (LOO‐CV) prediction errors and generalisation metrics. For each participant, model parameters were estimated from the remaining nine individuals and used to predict the left‐out trajectory. Prediction error is reported as the normalised sum of squared errors (SSE). The generalisation ratio (mean of LOO‐CV error/mean of participant fit error) of 1.495 shows the cohort model has ~50% higher error on held‐out participants compared to individualised fits.
**Figure S1**. Individual hormone profiles and cross‐correlations. Each row corresponds to a participant, numbered in the left axis. (A) Raw 24‐h plasma ACTH (blue) and CORT (red) concentrations for each of the 10 healthy participants. (B) Corresponding z‐scored hormone signals used for cross‐correlation analysis. (C) Time‐lagged cross‐correlation functions between ACTH and CORT showing negative lag values at the peak correlation, consistent with a temporal delay between ACTH and CORT.
**Figure S2**. Wavelet power of circadian‐detrended CORT signals for each of the 10 healthy participants. The colour scale represents wavelet power (yellow = high, dark blue = low) across time of day (x‐axis) and period (y‐axis, 10 to 300 min). Participants exhibit clear ultradian rhythmicity, with varying stability and strength of power bands throughout the 24‐h cycle.
**Figure S3**. Wavelet power of circadian‐detrended CORT signals for each of the 10 healthy participants. (A) Coloured lines show percentile curves (5th–95th) of wavelet power across time, illustrating the distribution of ultradian periodicities between 10 and 300 min. Peaks indicate dominant ultradian rhythms, with varying strength and sharpness across participants. (B) Total wavelet power (summed across time) plotted as a function of period (10–300 min) for each of the 10 healthy participants. Peaks represent dominant ultradian periodicities, which vary between individuals in both strength and frequency.
**Figure S4**. Bifurcation analysis of model with two delays. (A) Hopf bifurcation diagram for the original two‐delay model described by Malek et al., varying the delays *τ*_*a*_ and *τ*_*c*_. The shaded region indicates parameter combinations leading to oscillatory solutions. Since the qualitative dynamics were preserved across a range of delay combinations, we consolidated the two feedback delays into a single delay term in the simplified model to reduce complexity and the number of model parameters. (B) Intersection of Floquet multipliers with the imaginary axis, indicating a Hopf bifurcation for the 1‐delay model in the main text.
**Figure S5**. Two‐parameter bifurcation diagrams illustrating oscillatory regimes in the simplified HPA axis model across individuals. Each panel shows the region of self‐sustained oscillations as the blue‐shaded area varying with the feedback delay (𝜏) and basal CRH drive (𝑡). The length of the red vertical lines indicate the estimated CRH dynamic range for each individual model fit.
**Figure S6**. (A) Selected stressor timings (green dashed lines) shown relative to the model's circadian CRH drive (black) and the ultradian phases in the baseline cortisol output (red). (B, C) Examples of CORT responses (orange) to short stressor perturbations applied at different circadian and ultradian phases. Shaded areas show the change in area under the curve (AUC) relative to baseline (red) CORT signals, and arrows indicate the difference in peak CORT following the perturbation. The grey line represents the perturbed CRH signal.
**Figure S7**. Hormonal peak excursions following stressors, characterised by phase‐response heatmaps as a function of the circadian and ultradian phase. In both panels, the stressor duration is fixed at 100 min, while the magnitude of the stressor varies in the y‐axis. (A) reports the difference in peak concentration, whereas (B) reports the difference in AUC.
**Figure S8**. Computer simulations comparing model predictions under (A) constant CRH inputs versus oscillatory CRH inputs at periods of (B) 35 min, (C) 60 min and (D) 90 min, with CRH oscillation amplitudes fixed at 0.3 times the baseline in all cases. A rhythmic CRH drive at low and high baselines (outside the bifurcation region in Figure 4C) forced ACTH and CORT to follow the imposed CRH oscillatory period. At medium CRH levels however, ACTH and CORT preserve their intrinsic ultradian periodicity despite CRH oscillatory forcing at a different frequency, indicating robustness of the pituitary–adrenal pulse generator mechanism. As CRH periodicity approaches the lower bound (>90 min) of the period range observed in ACTH and CORT, the entrainment effect becomes apparent for low CRH, with period doubling oscillations emerging for intermediate CRH levels.
**Figure S9**. Dominant ultradian periods estimated from the mean wavelet power spectrum for each participant using fixed sliding window lengths of 480, 600, 720, 840, and 960 min, and the case where no normalisation was applied. The analysis was restricted to the physiologically supported ultradian period band of 50–250 min. Across window lengths, the estimated dominant periods remained broadly consistent, indicating that the identification of ultradian periodicity was not determined by the specific sliding window choice.
**Figure S10**. Cosinor analysis on circadian‐detrended cortisol time series (grey) show their corresponding best‐fitting cosinor models (colour) for each participant. The model fits capture the dominant ultradian oscillatory modes in the data.
**Figure S11**. Cosinor analysis adjusted values are shown across candidate ultradian periods (50–250 min) for each participant. Solid lines indicate model fits with one (blue) and two (orange) oscillatory mode components. Vertical dashed lines indicate the best‐fitting period in each case. While a dominant ultradian timescale is generally observed, multiple peaks and comparable fits across periods are present in some participants, reflecting variability in the data and limitations in resolving a unique periodicity in short, noisy time series.
**Figure S12**. Leave‐one‐out cross validation (LOO‐CV) analysis for model generalisability. (A) Distribution of normalised sum of squared error (SSE) for individual fits and LOO‐CV predictions. (B) Per‐participant comparison of prediction error between the LOO‐CV signal and the participant's best fit signal. (C) Generalisation ratio (LOO‐CV SSE/participant SSE). LOO‐CV errors are moderately higher than individual fits but remain comparable in magnitude, indicating that the model captures shared dynamics across participants.
**Figure S13**. Representative LOO‐CV predictions. Observed (dashed) and predicted (solid) ACTH and CORT profiles using cohort parameters.

## Data Availability

All data, metadata and detailed protocols associated to this study have been published elsewhere (see Methods). The computer code and data can be accessed from https://github.com/belindalombard/HPAAxisModel.
