# Supplementary material for: Regulation of ultradian pulsatility and stress responses in the human hypothalamic–pituitary–adrenal axis
Source: J Neuroendocrinol. 2026 Sep 8;38(9):e70264. doi: 10.1111/jne.70264 (PMC13553141; doi:10.1111/jne.70264)
Supplement: Supplementary file 1 [file JNE-38-e70264-s001.pdf]

Supplementary material

Model Development

We began with the model of Malek et al. (2015), where ACTH and CORT is described under delayed negative feedback from glucocorticoids. The following model equations (with some parameters renamed) govern Malek's model:

$$\frac{dA}{dt} = -\gamma_a A + h \frac{K_c^{m_a}}{K_c^{m_a} + C(t-\tau_1)^{m_a}}$$
$$\frac{dC}{dt} = -\gamma_c C + \alpha \frac{A(t-\tau_2)^{m_c}}{K_a^{m_c} + A(t-\tau_2)^{m_c}}$$

Key simplifications were made. First, we removed the parameter h. In Malek's model, h controls ACTH production, but in our framework this role is governed by an explicit hypothalamic drive $\Phi(t)$. The function $\Phi(t)$ captures the circadian modulation within the HPA axis and makes a separate scaling factor unnecessary. Unlike Malek's sinusoidal input, which represents SCN control with a fixed trigonometric function, we developed $\Phi(t)$ as a more flexible circadian drive that allows for an asymmetric profile, amplitude variation, and phase shifts.

We also combined the two delays τ_1 and τ_2 into a single effective delay τ, used to delay CORT. Both the feedback from CORT and the time it takes the adrenal glands to produce CORT after ACTH stimulation contribute to delays in the system. However, through bifurcation analysis with the two delays we found that using a single combined delay was enough to reproduce the same oscillatory behaviour as with two separate delays (Fig. S4A). This simplification makes the model easier to work with while still capturing the key ultradian dynamics.

Table S1. Model parameters.

Parameters		Selected boundaries		Fitting results	
Parameter	Description	Boundary	Boundary justification	Median	Mean $\pm$ std
m_c	Hill coefficient for ACTH-driven CORT production (AU)	(2, 5)	Allows for nonlinear saturation	3.23	3.40 ± 0.62
γ_c	Degradation rate of CORT (min^{-1})	(0.0231, 0.0693)	Corresponds to measured half-life of cortisol (10–30 min)	0.0066	0.0072 ± 0.0016
K_a	ACTH receptor half-saturation constant ($pmol/L$)	(1,25)	Based on observed ACTH data	8.32	9.93 ± 4.86
α	Maximal rate of ACTH-induced CORT production (nM/min)	(1, 100)	Broad range to allow variation in adrenal sensitivity	20.44	20.78 ± 2.84
γ_a	Degradation rate of ACTH (min^{-1})	(0.00578, 0.00991)	Matches measured half-life of ACTH (70–120 min	0.026	0.031 ± 0.0093
m_a	Hill coefficient for CORT feedback (AU)	(2, 5)	Consistent with cooperative receptor binding	3.32	3.44 ± 0.443
K_c	CORT receptor half-saturation constant ($nmol/L$)	(8, 150)	Based on observed CORT data	41.65	39.82 ± 6.026
τ	Feedback delay from CORT to ACTH (min)	(1, 40)	Physiological delays of 10–30 min in glucocorticoid feedback	22.43	22.19 ± 3.17
λ_s	Circadian modulation strength (AU)	(0, 20)	Controls base level of $\Phi(t)$	2.78	2.94 ± 0.697
λ_a	Baseline amplitude of CRH drive (AU)	(0, 20)	Shapes circadian modulation strength	8.22	9.32 ± 3.87
t_s	Circadian phase shift (min)	(-1440, 1440)	Minutes of the day	12.68	30.17 ± 51.35
σ	Asymmetry of circadian drive (AU)	(0, 1)	Shapes circadian profile symmetry	0.78	0.73 ± 0.21

Table S2. Leave-one-out cross-validation (LOO-CV) prediction errors and generalisation metrics. For each participant, model parameters were estimated from the remaining nine individuals and used to predict the left-out trajectory. Prediction error is reported as the normalised sum of squared errors (SSE). The generalisation ratio (mean of LOO-CV error/mean of participant fit error) of 1.495 shows the cohort model has ~50% higher error on held-out participants compared to individualised fits.

Metric	Participant fit (individualised)	Cohort model (LOO-CV)	Interpretation
**Mean SSE ± SD**	53.6930 ± 16.6567	83.8851 ± 29.8666	Similar means indicate good generalisation
**Median SSE**	58.0750	76.8448	Central tendency measure
**Generalisation ratio**	1.000 (reference)	1.562	How much worse is LOO-CV signal?
**Participants with improved fit**	-	1/10 (10%)	LOO-CV error lower than participant fit
**Max error increase**	-	180.9%	Worst-case generalisation penalty

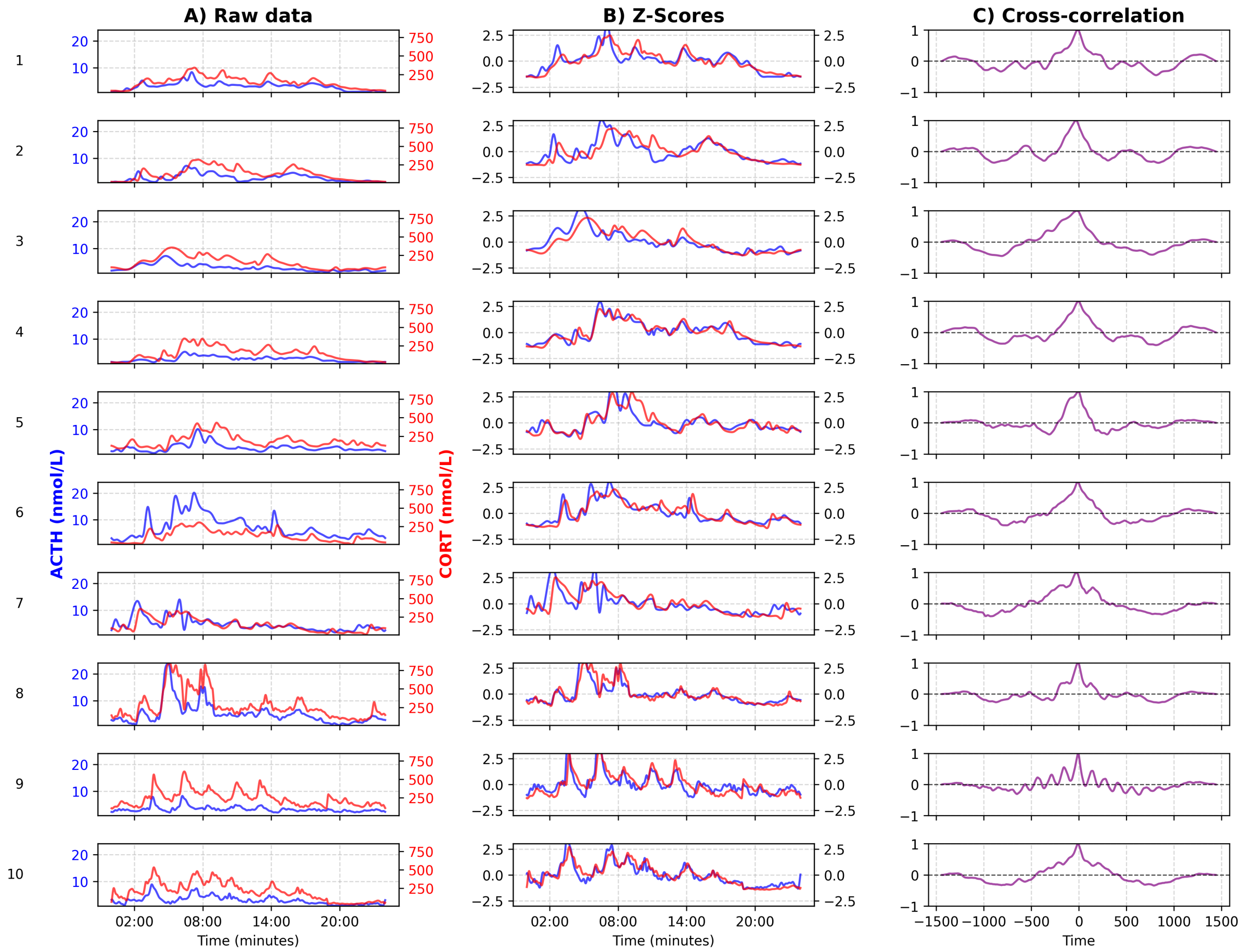

Figure S1. Individual hormone profiles and cross-correlations. Each row corresponds to a participant, numbered in the left axis. **A)** Raw 24-hour plasma ACTH (blue) and CORT (red) concentrations for each of the ten healthy participants. **B)** Corresponding z-scored hormone signals used for cross-correlation analysis. **C)** Time-lagged cross-correlation functions between ACTH and CORT showing negative lag values at the peak correlation, consistent with a temporal delay between ACTH and CORT.

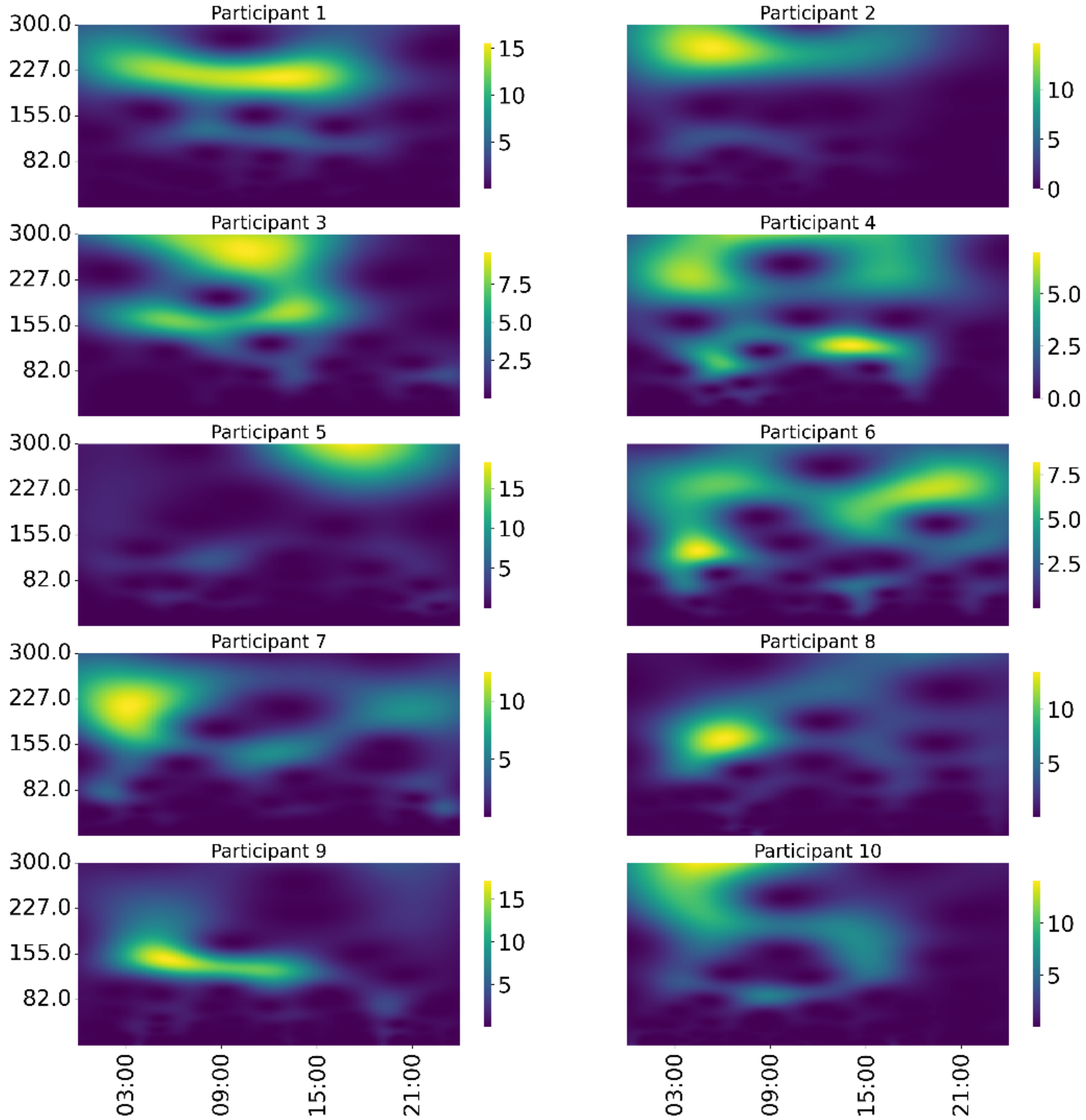

Figure S2. Wavelet power of circadian-detrended CORT signals for each of the ten healthy participants. The colour scale represents wavelet power (yellow = high, dark blue = low) across time of day (x-axis) and period (y-axis, 10 to 300 min). Participants exhibit clear ultradian rhythmicity, with varying stability and strength of power bands throughout the 24-hour cycle.

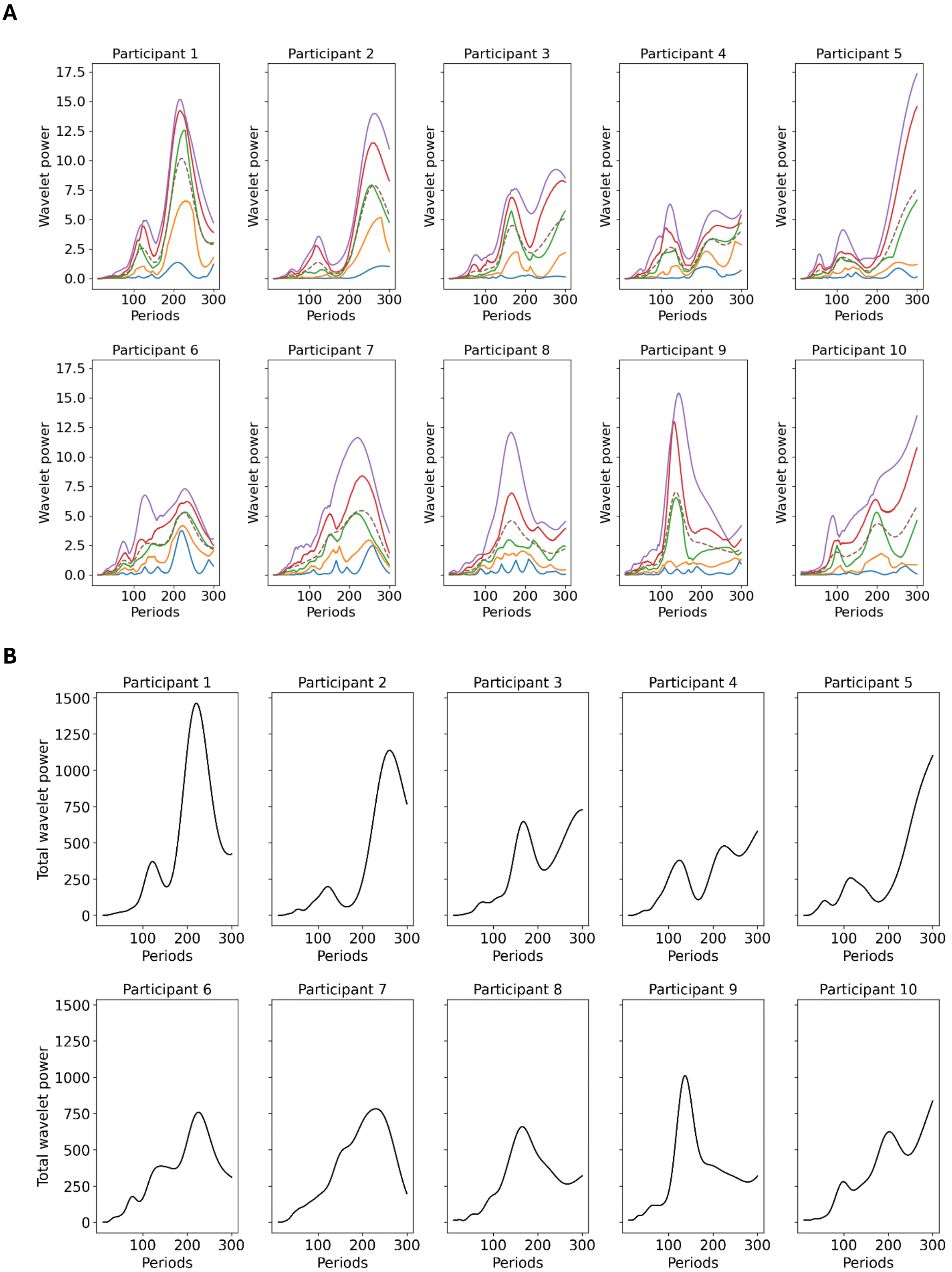

Figure S3. Wavelet power of circadian-detrended CORT signals for each of the ten healthy participants. **A)** Coloured lines show percentile curves (5th–95th) of wavelet power across time, illustrating the distribution of ultradian periodicities between 10 and 300 minutes. Peaks indicate dominant ultradian rhythms, with varying strength and sharpness across participants. **B)** Total wavelet power (summed across time) plotted as a function of period (10–300 min) for each of the ten healthy participants. Peaks represent dominant ultradian periodicities, which vary between individuals in both strength and frequency.

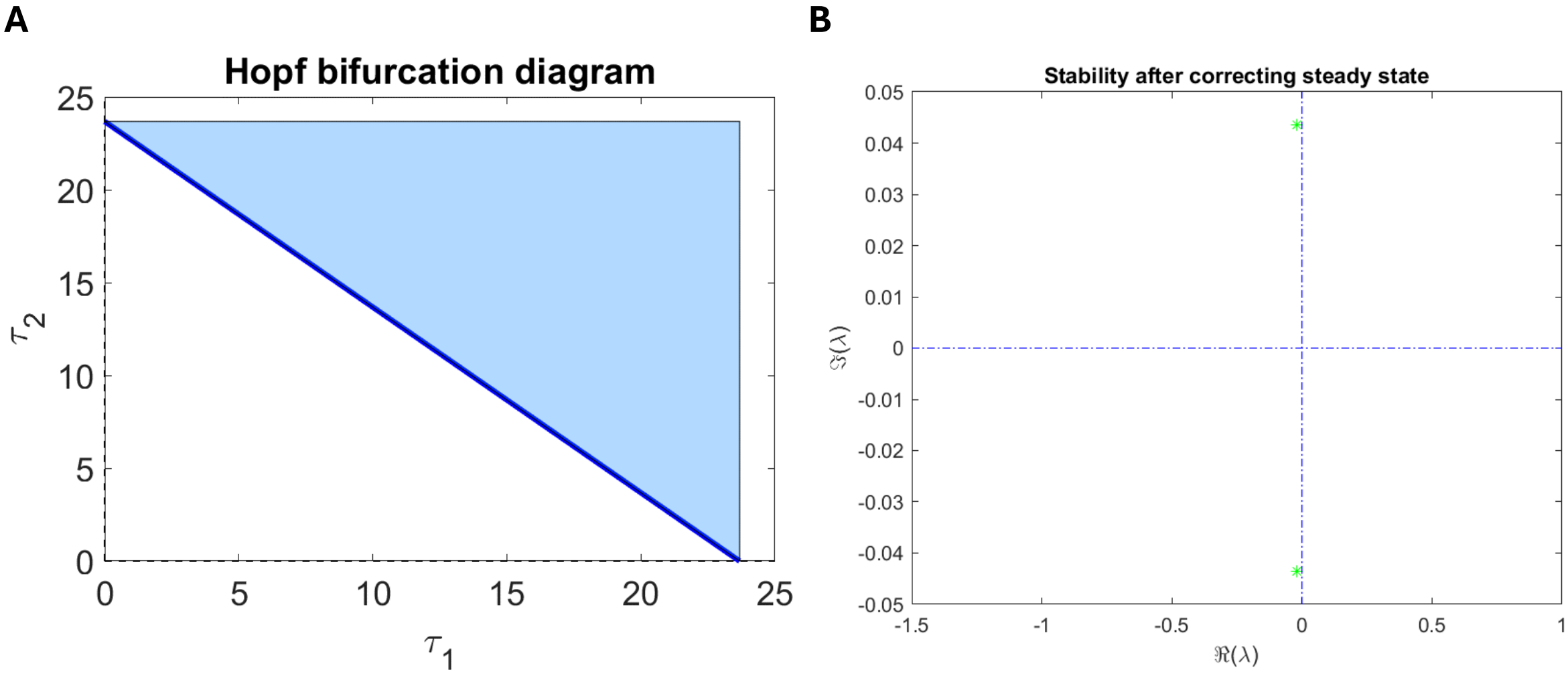

Figure S4. Bifurcation analysis of model with two delays. **A)** Hopf bifurcation diagram for the original two-delay model described by Malek (2015), varying the delays τ_a and τ_c. The shaded region indicates parameter combinations leading to oscillatory solutions. Since the qualitative dynamics were preserved across a range of delay combinations, we consolidated the two feedback delays into a single delay term in the simplified model to reduce complexity and the number of model parameters. **B)** Intersection of Floquet multipliers with the imaginary axis, indicating a Hopf bifurcation for the 1-delay model in the main text.

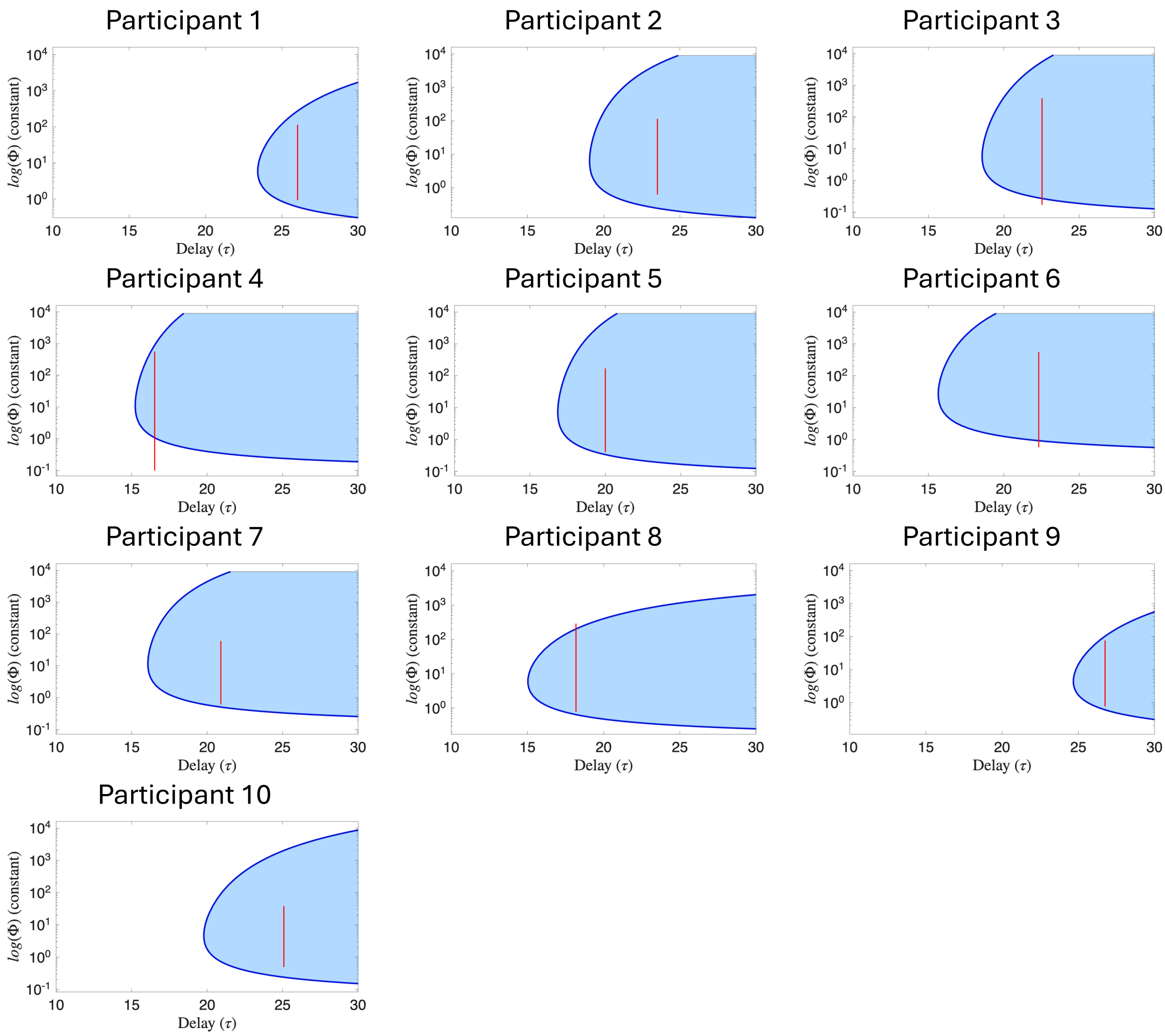

Figure S5. Two-parameter bifurcation diagrams illustrating oscillatory regimes in the simplified HPA axis model across individuals. Each panel shows the region of self-sustained oscillations as the blue-shaded area varying with the feedback delay (τ) and basal CRH drive ($\Phi(t)$). The length of the red vertical lines indicate the estimated CRH dynamic range for each individual model fit.

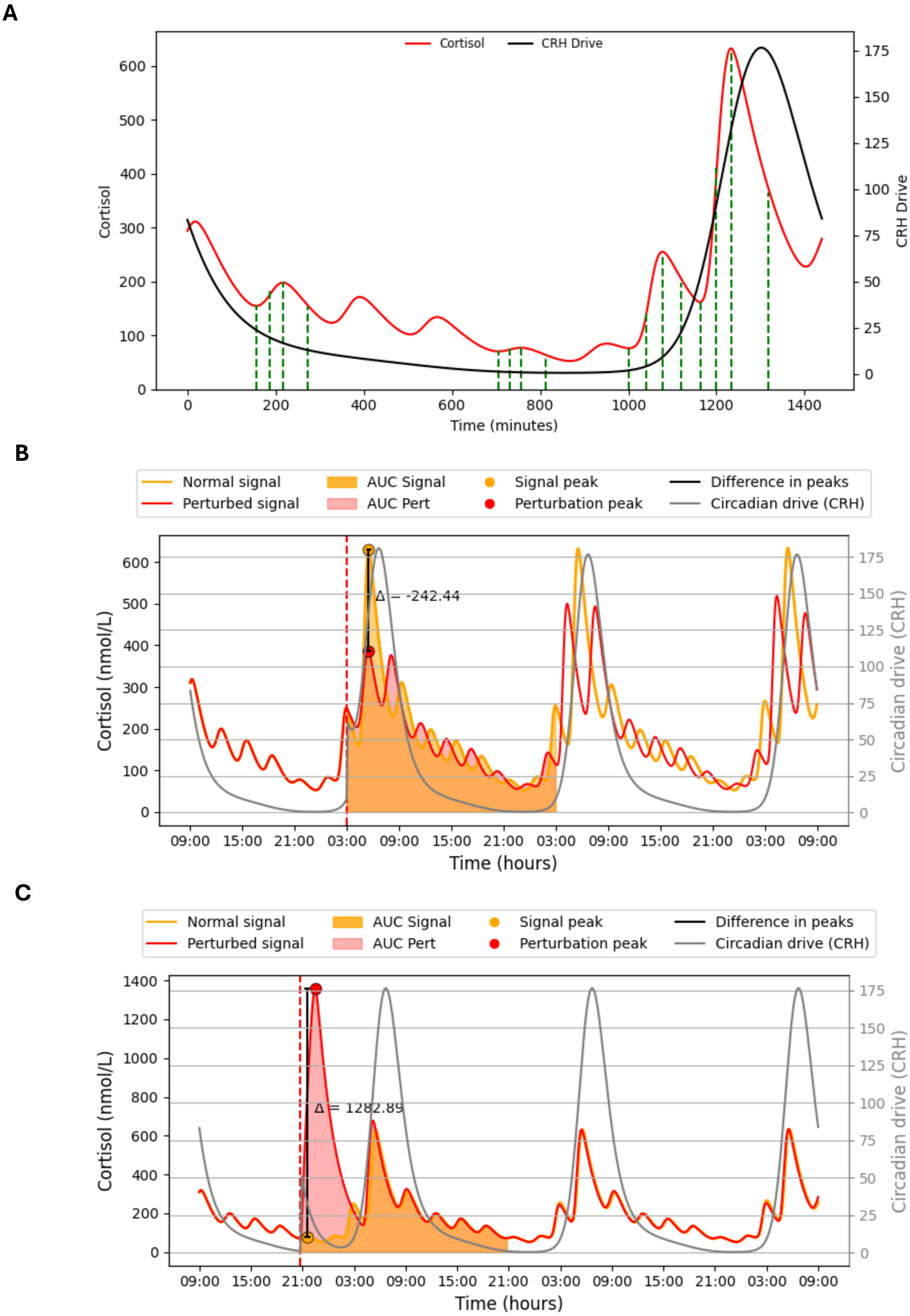

Figure S6. A) Selected stressor timings (green dashed lines) shown relative to the model's circadian CRH drive (black) and the ultradian phases in the baseline cortisol output (red). **B-C)** Examples of CORT responses (orange) to short stressor perturbations applied at different circadian and ultradian phases. Shaded areas show the change in area under the curve (AUC) relative to baseline (red) CORT signals, and arrows indicate the difference in peak CORT following the perturbation. The grey line represents the perturbed CRH signal.

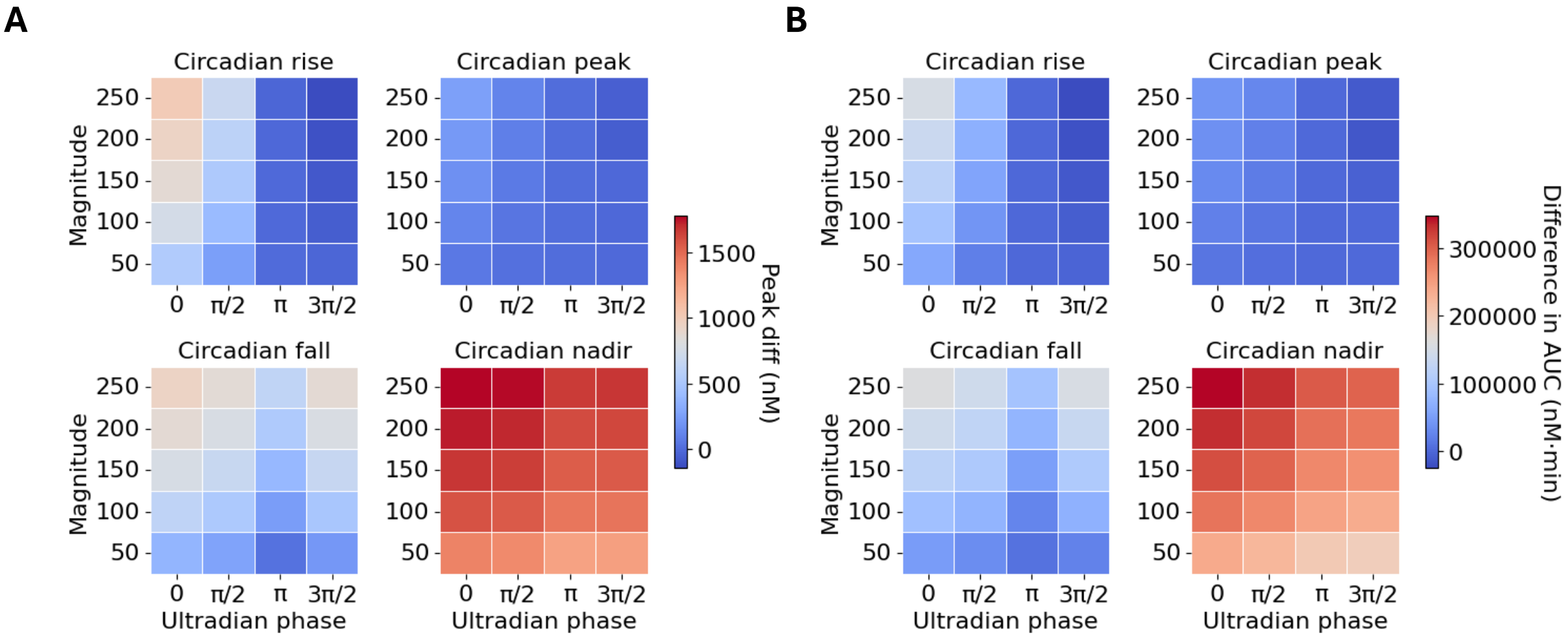

Figure S7. Hormonal peak excursions following stressors, characterised by phase-response heatmaps as a function of the circadian and ultradian phase. In both panels, the stressor duration is fixed at 100 minutes, while the magnitude of the stressor varies in the y-axis. **A)** reports the difference in peak concentration, whereas **B)** reports the difference in AUC.

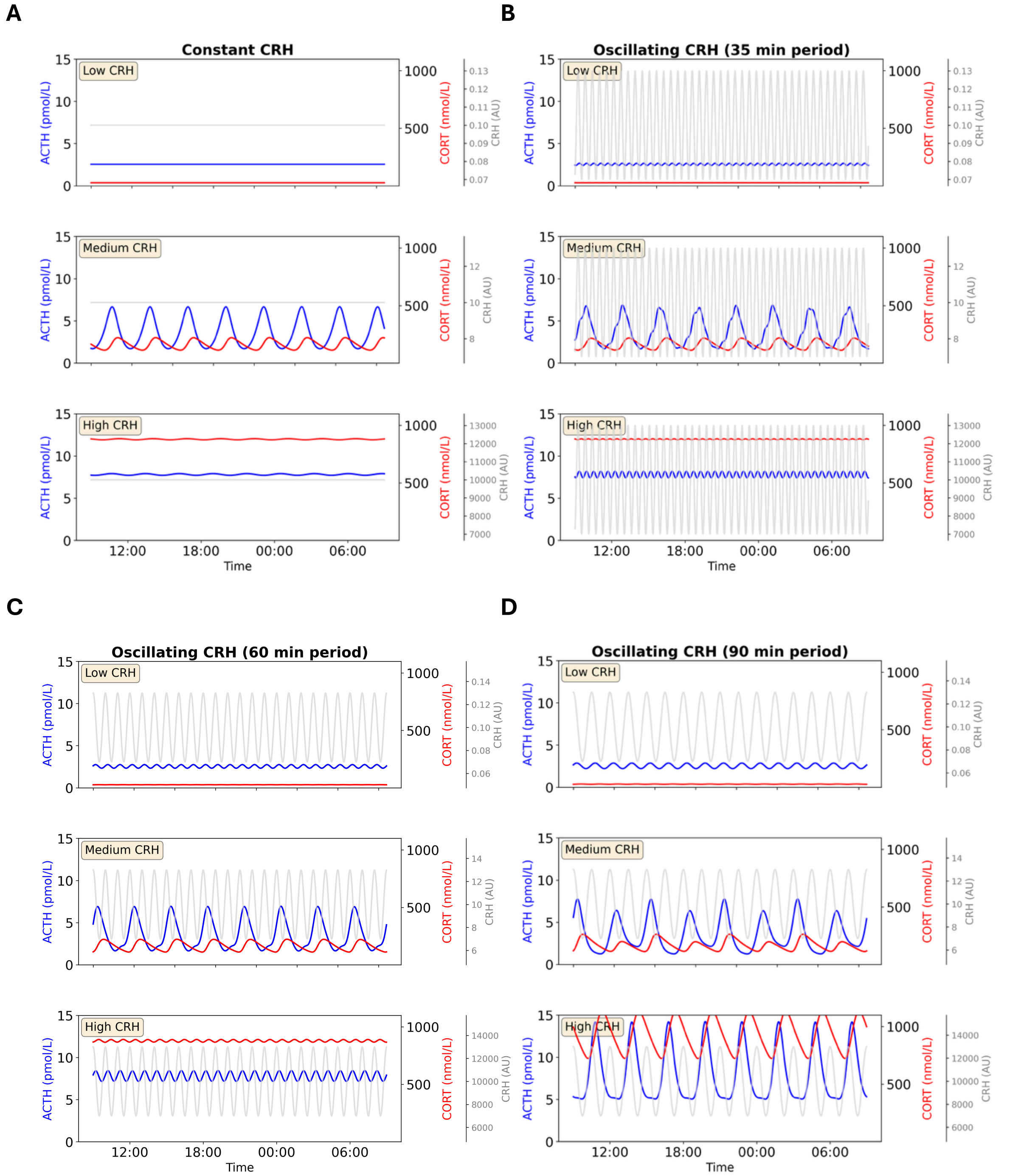

Figure S8. Computer simulations comparing model predictions under **A)** constant CRH inputs versus oscillatory CRH inputs at periods of **B)** 35 min, **C)** 60 min and **D)** 90 min, with CRH oscillation amplitudes fixed at 0.3 times the baseline in all cases. A rhythmic CRH drive at low and high baselines (outside the bifurcation region in Fig. 4C) forced ACTH and CORT to follow the imposed CRH oscillatory period. At medium CRH levels however, ACTH and CORT preserve their intrinsic ultradian periodicity despite CRH oscillatory forcing at a different frequency, indicating robustness of the pituitary–adrenal pulse generator mechanism. As CRH periodicity approaches the lower bound (>90 min) of the period range observed in ACTH and CORT, the entrainment effect becomes apparent for low CRH, with period doubling oscillations emerging for intermediate CRH levels.

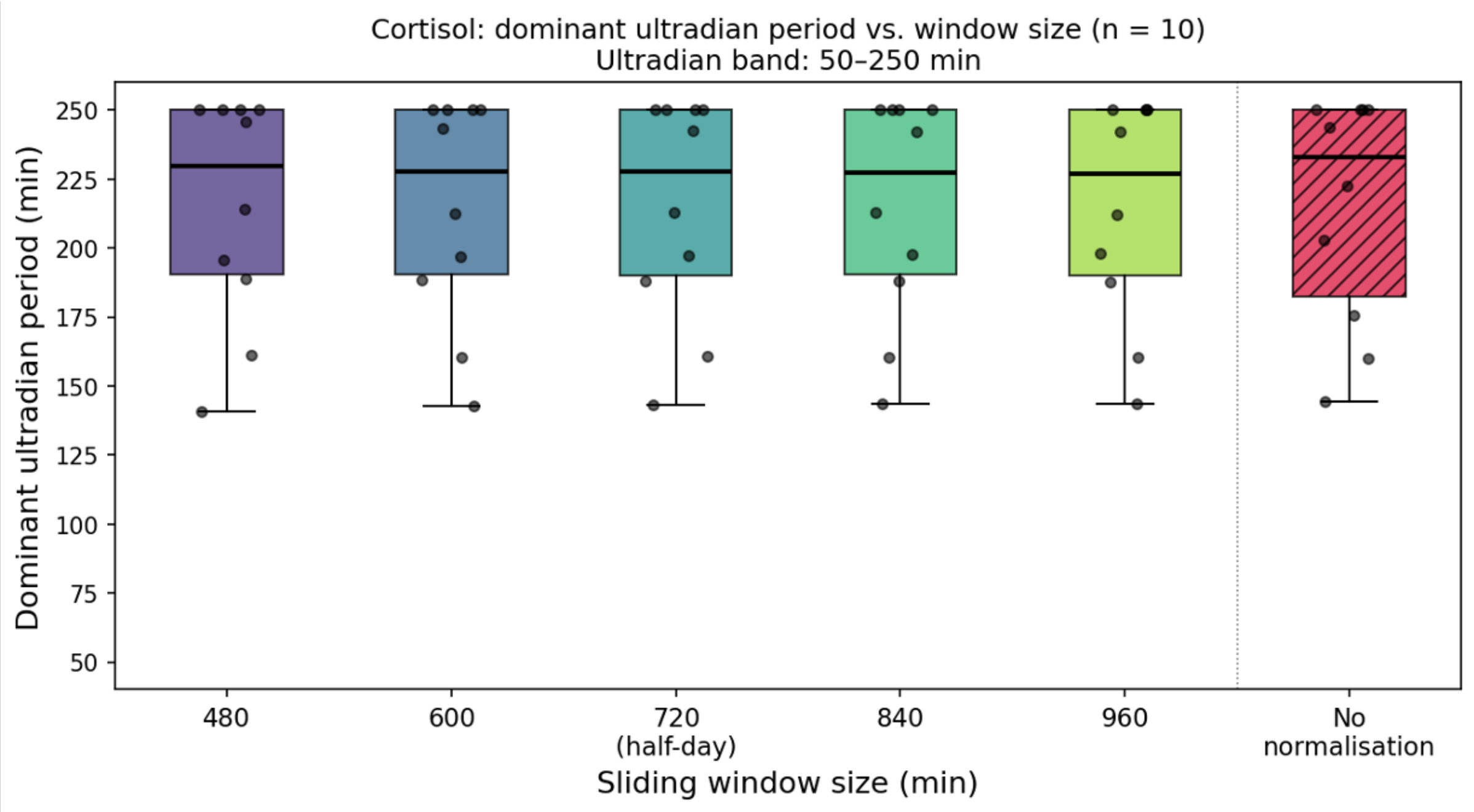

Figure S9. Dominant ultradian periods estimated from the mean wavelet power spectrum for each participant using fixed sliding window lengths of 480, 600, 720, 840, and 960 min, and the case where no normalisation was applied. The analysis was restricted to the physiologically supported ultradian period band of 50–250 min. Across window lengths, the estimated dominant periods remained broadly consistent, indicating that the identification of ultradian periodicity was not determined by the specific sliding window choice.

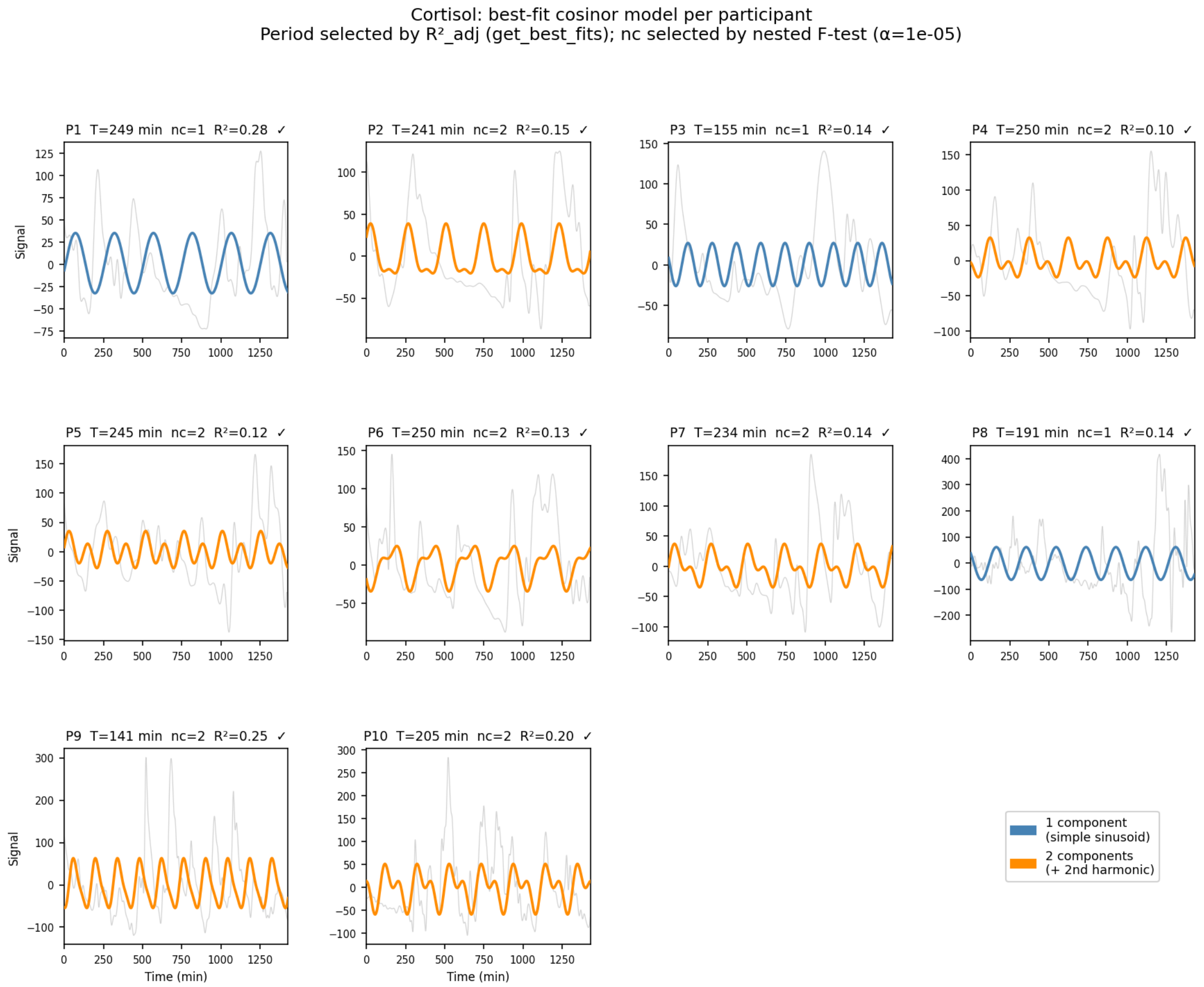

Figure S10. Cosinor analysis on circadian-detrended cortisol time series (grey) show their corresponding best-fitting cosinor models (colour) for each participant. The model fits capture the dominant ultradian oscillatory modes in the data.

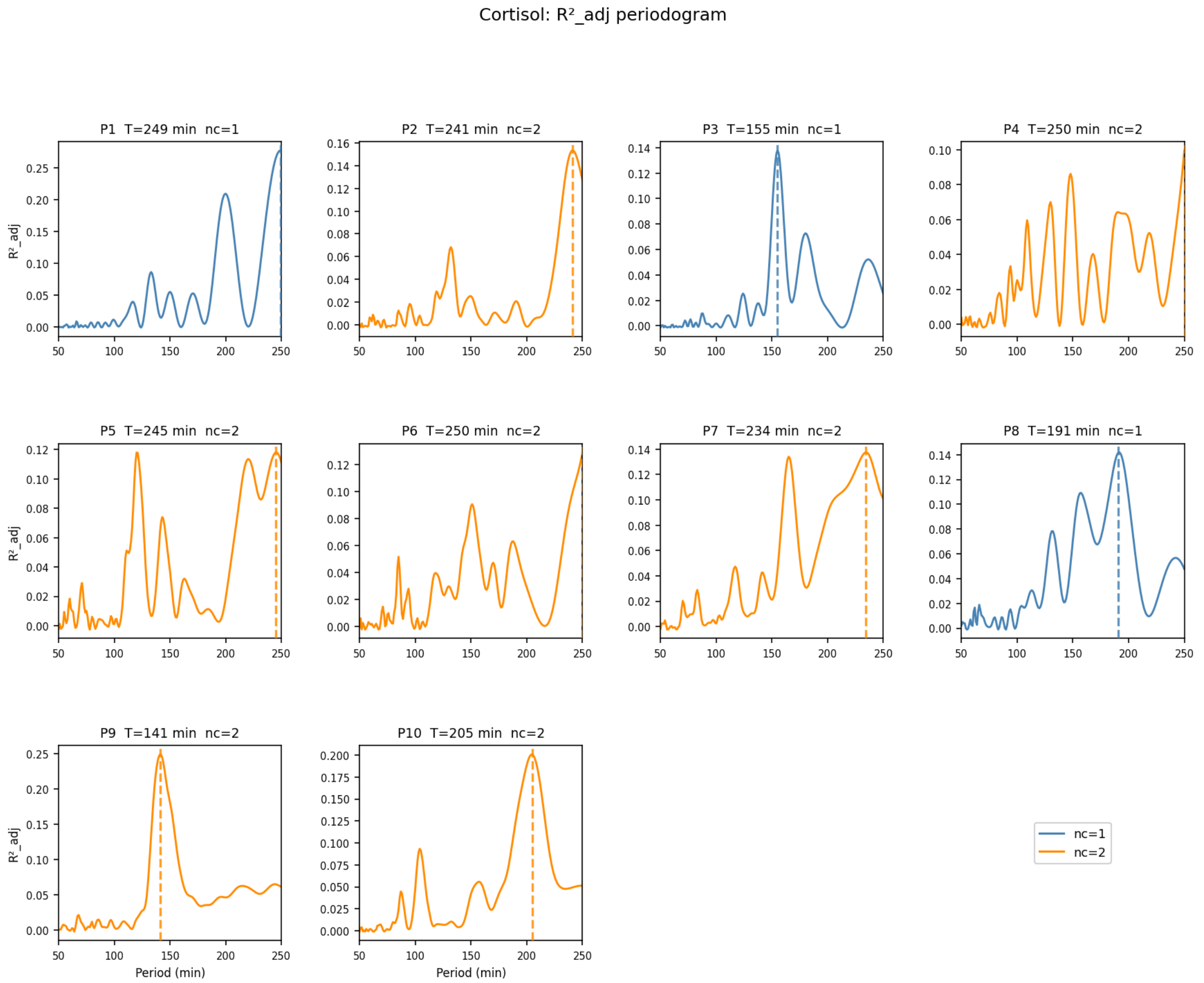

Figure S11. Cosinor analysis adjusted R^2 values are shown across candidate ultradian periods (50–250 min) for each participant. Solid lines indicate model fits with one (blue) and two (orange) oscillatory mode components. Vertical dashed lines indicate the best-fitting period in each case. While a dominant ultradian timescale is generally observed, multiple peaks and comparable fits across periods are present in some participants, reflecting variability in the data and limitations in resolving a unique periodicity in short, noisy time series.

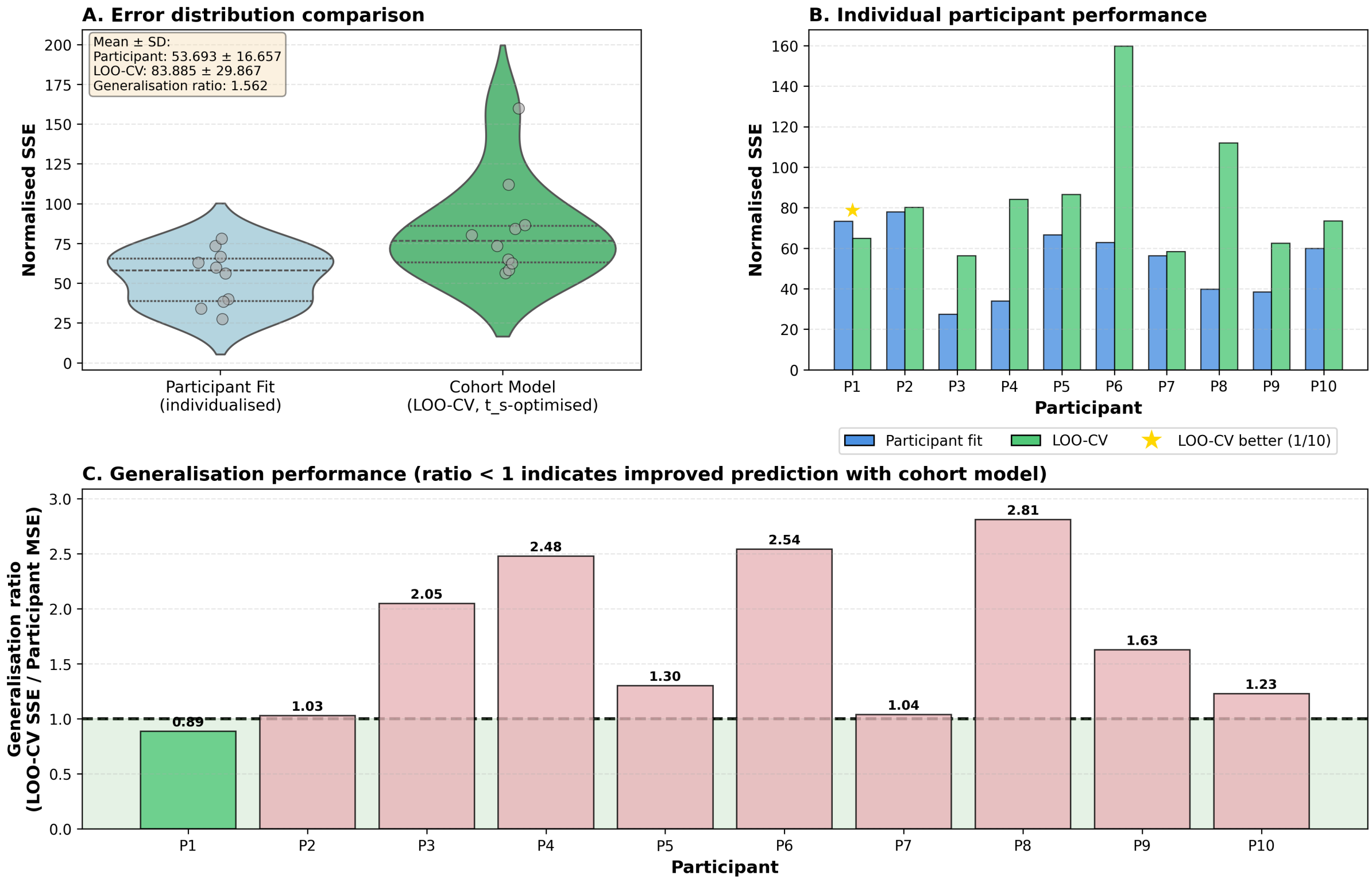

Figure S12. Leave-one-out cross validation (LOO-CV) analysis for model generalisability. **A)** Distribution of normalised sum of squared error (SSE) for individual fits and LOO-CV predictions. **B)** Per-participant comparison of prediction error between the LOO-CV signal and the participant's best fit signal. **C)** Generalisation ratio (LOO-CV SSE / participant SSE). LOO-CV errors are moderately higher than individual fits but remain comparable in magnitude, indicating that the model captures shared dynamics across participants.

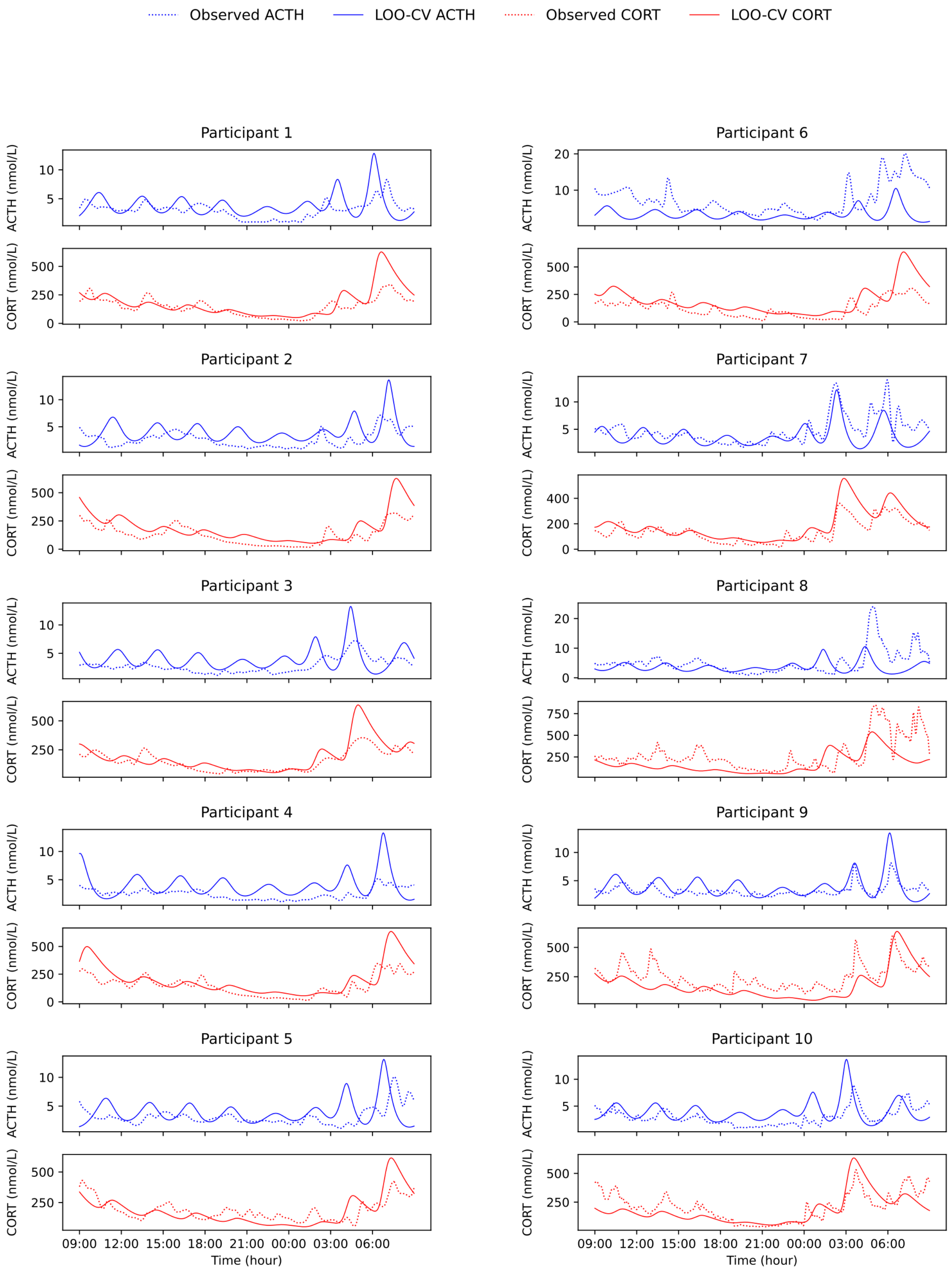

Figure S13. Representative LOO-CV predictions. Observed (dashed) and predicted (solid) ACTH and CORT profiles using cohort parameters.
